# Global impact of Occupational Noise-Induced Hearing Loss (ONIHL): trends, gender disparities, and future projections: 1990–2036

**DOI:** 10.3389/fgwh.2025.1584639

**Published:** 2025-07-22

**Authors:** Dandan Liu, Huixia Ji, Ye Chen, Wenying Li

**Affiliations:** Department of Occupational Disease, Nanjing Prevention and Treatment Center for Occupational Diseases, Nanjing, Jiangsu, China

**Keywords:** ONIHL, GBD, SDI, female, YLDs rate

## Abstract

**Background:**

The burden of Occupational Noise-Induced Hearing Loss (ONIHL) is escalating globally, presenting significant challenges to society and healthcare systems. This study aims to provide a comprehensive assessment of the global burden of ONIHL from 1990 to 2021, analyze these impacts by gender, and project the future burden of ONIHL over the next fifteen years.

**Methods:**

The data were sourced from the Global Burden of Disease (GBD) study conducted in 2021. A Joinpoint regression model was employed to calculate the annual percentage change (APC) in ONIHL Years Lived with Disability (YLDs) rate, and a decomposition analysis was utilized to quantify the influences of age structure, population growth, and epidemiological changes on the global burden of ONIHL. Additionally, predictions of future YLDs rate trends were made using Bayesian Age-Period-Cohort (BAPC) and Autoregressive Integrated Moving Average (ARIMA) models.

**Results:**

The global rate of ONIHL YLDs escalated from 3,838,055 person-years in 1990 to 7,847,445 person-years in 2021, with an age-standardized YLDs rate experiencing a 23% increase. Males exhibited a higher number and rate of YLDs than females, however, the growth rate for females was greater, with Estimated Annual Percentage Changes (EAPCs) recorded at 0.42 (0.41–0.43) for females and 0.11 (0.09–0.12) for males, respectively, and Average Annual Percentage Changes (AAPCs) of 0.44 (95% CI: 0.43–0.45) for females compared to 0.13 (95% CI: 0.12–0.14) for males. In 2021, the YLDs rate decreased as the Socio-Demographic Index (SDI) increased across 224 countries, indicating a concentration of the burden in countries with a medium SDI. Between 1990 and 2021, the incidence of ONIHL among females exhibited an upward trend in most countries, whereas among males, it predominantly reflected a downward trend. The decomposition analysis revealed that population growth was the primary factor contributing to the increase in YLDs. Projections indicate that by 2036, the YLDs rate for ONIHL will reach 103.45 per 100,000 in males and 74.19 per 100,000 in females.

**Conclusion:**

The global burden of ONIHL is rising at a concerning rate, particularly in countries with a medium SDI and among females. Therefore, it is imperative to implement targeted health education, regular screenings, and accessible hearing protection measures to mitigate the risks associated with ONIHL, specifically for females.

## Introduction

In 2021, age-related and other hearing loss resulted in 44·5 million (95% UI 30·7–62·0) Years Lived with Disability (YLDs). It was the fourth-ranked cause of YLDs in 2021 for non-communicable diseases (1). Hearing loss is recognized as the most prevalent sensory impairment worldwide, with occupational noise exposure identified as an important contributing factor. Occupational Noise-Induced Hearing Loss (ONIHL) poses a substantial public health challenge globally. Research demonstrates that individuals working in sectors such as construction, manufacturing, mining, agriculture, utilities, transportation, military service, and music are at the highest risk for ONIHL (2). Although ONIHL does not directly result in premature mortality, it can lead to considerable disability and associated issues. It may hinder effective communication, resulting in social stress, emotional distress, diminished self-esteem, identity crises, and weakened interpersonal relationships (3). What's more, studies indicate that exposure to high levels of noise can trigger physiological responses within the autonomic nervous and endocrine systems, resulting in enhanced secretion of stress hormones. This physiological reaction correlates with increased risks of hypertension, coronary heart disease, and stroke (4, 5). The ongoing discourse surrounding gender disparities in noise-induced hearing loss is particularly relevant, given the growing prevalence of women's exposure to various occupational noise levels (6). However, there is a noticeable lack of comprehensive information pertaining to the epidemiological characteristics and factors influencing hearing loss among females in the workforce. Most research on occupational noise exposure predominantly involves male participants (7), with some studies exclusively focusing on this demographic, potentially reflecting the higher percentage of men in noisy work environments (8). Thus, there is a pressing need for an in-depth epidemiological examination of gender differences associated with hearing loss resulting from occupational noise exposure.

This study seeks to evaluate the global disease burden of hearing loss attributed to ONIHL from 1990 to 2021. It further investigates factors such as sex, age, and the SDI to address gaps identified in the Global Burden of Disease (GBD) reports. By analyzing variations in YLDs across different sexes, ages, and SDI categories over the last 32 years, this research offers essential insights for the formulation of occupational noise safety initiatives worldwide.

## Data sources and definitions

The GBD database employs comparative risk assessment methodologies to estimate mortality rates attributable to various risk factors, along with years of life lost (YLLs), YLDs and disability-adjusted life years (DALYs). This comprehensive analysis encompasses 87 risk factors across 204 countries and regions worldwide, covering data from 1990 to 2021 (9). Specific information regarding age-related and other forms of hearing loss, including YLD numbers, YLD rates, and their age-standardized rates (ASR) in Global from 1990 to 2021, was sourced from the Institute for Health Metrics and Evaluation (https://vizhub.healthdata.org/gbd-results/). The waiver of knowledgeable consent for the GBD study has received approval from the Institutional Review Board at the University of Washington.

DALYs encompass YLLs due to premature mortality and YLDs resulting from illness. Hearing loss associated with occupational noise does not typically lead to premature death, which contributes to lower YLL figures, thus, YLDs are identified as the primary measure of disease burden. YLDs are computed by multiplying the prevalence of a condition by its corresponding disability weight, which reflects the severity of that condition compared to all other health states (10). Disability weights range from 0, symbolizing perfect health, to 1, representing death. Hearing loss is defined as a reduction in auditory capability greater than 20 dB in the better ear, calculated as the average across four frequencies (500–4,000 Hz) (11). The GBD study characterizes hearing loss as the average minimum sound level detectable by an individual's better-hearing ear, assessed through the pure-tone average hearing threshold at 0.5, 1, 2, and 4 kHz. It categorizes hearing loss into seven severity levels, ranging from normal (0–19 dB) to important (≥95 dB), while also considering the presence of tinnitus (12). Occupational noise exposure is defined as the percentage of the population that has encountered noise levels of 85 decibels or greater in the workplace, based on population distributions across 17 economic sectors (13).

GBD 2019 used a diverse array of data sources, including censuses, surveys, registries, surveillance, and scientific literature, to assess the prevalence and impact of various risk factors associated with hearing loss (12). To mitigate challenges related to data gaps and inconsistencies, GBD 2019 implemented a range of statistical techniques. These included employing random forest algorithms for imputing missing values, applying hierarchical Bayesian regression models to smooth and refine raw data, and using causal diagram models to evaluate the interrelationships among risk factors (12). Building on these methodologies and the resulting data, we developed our models to investigate trends and determinants of hearing loss across different geographic regions and time periods.

Socio-Demographic Index (SDI) is an aggregative metric that measures the development of a country or region, combining data on the total fertility rate for females under 25, the average level of education of females aged 15 and over, and per capita income, which is closely related to the residents' health status. The value range of SDI is (0, 1), with higher scores representing higher per capita income and education levels, but lower fertility rates. Based on the SDI value, countries or regions are categorized into low SDI, low-middle SDI, middle SDI, high-middle SDI, and high SDI (14).

## Statistical analysis

This study provides insights into the YLDs and ASRs of ONIHL globally and according to SDI, stratified by age and gender, accompanied by 95% uncertainty intervals (UIs). The 95% UI reflects the range of potential values around an estimate, indicating a 95% confidence that the true value falls within this range, leaving a 5% probability that it lies outside. We calculated 95% UIs by employing 1,000 samples from the posterior distribution at each stage of the estimation process, using the 2.5th and 97.5th percentiles of the ordered values (9)**.**

Joinpoint analysis was used to discern trends in ONIHL YLDs from 1990 to 2021. As introduced by Kim in 2000 (15), this method allows for the segmentation of longitudinal data through piecewise regression, enabling the identification of statistically major trends within these segments. Regression analysis was conducted on the natural logarithm of YLD rates across various segments, followed by the calculation of the annual percentage change (APC) and its corresponding 95% confidence interval (CI) for each period. The global trend was represented by the Average Annual Percentage Changes (AAPCs). Statistical significance for both APC and AAPC was established through non-overlapping 95% CIs and a *p*-value of less than 0.05, compared to the null hypothesis of no variation. Besides, we employed the decomposition method developed by Das Gupta to assess the impacts of age structure, population growth, and epidemiological changes on the global burden of asbestosis (16)**.**

The autoregressive integrated moving average (ARIMA) model was used to forecast the future trends of ONIHL yield rates over the next fifteen years. The model is represented as ARIMA (p, d, q), where p denotes the autoregressive order, d indicates the number of differences, and q represents the moving average order (17). The differencing technique was applied to convert non-stationary data into stationary data. Subsequently, the autocorrelation function (ACF) and partial autocorrelation function (PACF) were plotted to assess the stationarity of the sequence following differencing. The auto.Arima () function was employed to identify the optimal model based on the Akaike information criterion (AIC). This function is adept at analyzing various ARIMA models for univariate time series data, exploring potential models in accordance with specified constraints, and determining the most suitable model (18, 19). The normality of the residuals was evaluated through QQ plots, and ACF and PACF plots. The Ljung–Box test for white noise was used to examine the presence of serial correlations in the residuals. The predictive accuracy of the ARIMA models was assessed using metrics such as mean error (ME), root mean squared error (RMSE), mean absolute error (MAE), mean percentage error (MPE), mean absolute percentage error (MAPE), and mean absolute scaled error (MASE).

Additionally, we employed the Bayesian Age-Period-Cohort (BAPC) model to project the future trends of the global burden of ONIHL YLDs rates for both females and males, using this as an external method to validate the accuracy of the ARIMA model. The BAPC approach has been widely used in epidemiological studies to analyze temporal trends in chronic diseases and predict future disease burdens (20, 21).

Joinpoint analysis was performed using Joinpoint Regression software version 5.3 (Statistical Research and Applications Branch of the National Cancer Institute, USA). The ARIMA analysis and associated visualizations were conducted with R software version 4.2 (R core team), using the “forecast,” “tseries,” and “ggplot2” packages, and the BAPC analysis was implemented using the R software packages “INLA” and “BAPC”. A *p*-value of less than 0.05 was deemed statistically important.

## Results

### Burden of YLDs of ONIHL during 1990–2021

#### Global

From 1990 to 2021, the number of YLDs due to occupational noise demonstrated a major increase, rising from 3,838,055 person-years (95% UI: 2,630,899–5,373,293) to 7,847,445 person-years (95% UI: 5,313,648–10,980,789). The age-standardized YLDs rate increased from 84.28 per 100,000 person-years (95% UI: 57.62–118.17) to 91.12 (95% UI: 61.98–127.2), representing a 23% rise. Analyzing gender differences, male YLDs rose from 2,427,796 (95% UI: 1,658,223–3,398,390) to 4,779,975 (95% UI: 3,229,199–6,666,481), with the age-standardized YLDs rate increasing from 108.98 (95% UI: 74.17–152.26) to 113.35 (95% UI: 76.93–157.86). In contrast, female YLDs grew from 1,410,259 years (95% UI: 963,823–1,986,866) to 3,067,470 years (95% UI: 2,087,477–4,314,308), while the age-standardized YLDs rate rose from 60.98 (95% UI: 41.37–85.93) to 69.87 (95% UI: 47.67–97.95). By comparing the data from 2021 to 1990, the global challenge of hearing loss resulting from noise exposure has become increasingly important for both genders. Although the burden of ONIHL is notably higher in males, the rate of increase for females has outpaced that of males, with EAPC of 0.42 (0.41–0.43) for females compared to 0.11 (0.09–0.12) for males (Table 1).

**Table 1:** Global and SDI quintile regions cases and age-standardised YLDs rates of ONIHL from 1990 to 2021.

Location	1990						2021						EAPC (95%CI)		
	Total		Female		Male		Total		Female		Male		Total	Female	Male
	Cases (95%UI)	ASR per 100000 (95%UI)	Cases (95%UI)	ASR per 100000 (95%UI)	Cases (95%UI)	ASR per 100000 (95%UI)	Cases (95%UI)	ASR per 100000 (95%UI)	Cases (95%UI)	ASR per 100000 (95%UI)	Cases (95%UI)	ASR per 100000 (95%UI)			
Global	3,838,055 (2,630,899, 5,373,293)	84.28 (57.62, 118.17)	1,410,259 (963,823, 1,986,866)	60.98 (41.37, 85.93)	2,427,796 (1,658,223, 3,398,390)	108.98 (74.17, 152.26)	7,847,445 (5,313,648, 10,980,789)	91.12 (61.98, 127.2)	3,067,470 (2,087,477, 4,314,308)	69.87 (47.67, 97.95)	4,779,975 (3,229,199, 6,666,481)	113.35 (76.93, 157.86)	0.23 (0.21, 0.25)	0.42 (0.41, 0.43)	0.11 (0.09, 0.12)
High SDI	445,410 (302,453, 629,366)	43.46 (29.37, 61.72)	161,384 (111,494, 228,400)	29.97 (20.57, 42.46)	284,026 (190,657, 404,010)	58.61 (39.33, 83.11)	734,617 (495,089, 1,039,611)	45.56 (30.66, 64.63)	271,228 (185,740, 385,777)	33.07 (22.51, 47.02)	463,389 (309,870, 658,099)	58.4 (38.98, 82.57)	0.15 (0.12, 0.18)	0.33 (0.32, 0.35)	−0.02 (−0.07, 0.03)
High-middle SDI	851,449 (577,126, 1,196,402)	79.32 (53.63, 111.58)	344,431 (231,443, 482,560)	61.61 (41.3, 86.57)	507,018 (345,188, 715,207)	99.49 (67.79, 139.63)	1,654,261 (1,112,237, 2,342,966)	91.4 (61.49, 127.84)	692,363 (463,007, 980,935)	74.33 (49.73, 104.68)	961,899 (646,587, 1,359,363)	109.65 (73.69, 153.14)	0.49 (0.47, 0.5)	0.63 (0.61, 0.64)	0.35 (0.33, 0.37)
Middle SDI	1,424,978 (972,881, 1,998,624)	107.5 (72.62, 151.54)	542,415 (367,770, 763,356)	82.71 (55.74, 116.31)	882,563 (602,765, 1,235,248)	132.46 (89.76,185.65)	3,014,042 (2,027,104, 4,241,934)	107.22 (72.41, 150.41)	1,208,670 (808,429, 1,709,208)	84.26 (56.74, 118.69)	1,805,372 (1,215,604, 2,533,879)	131.1 (88.43, 183.79)	−0.01(−0.02, 0)	0.07 (0.06, 0.08)	−0.04(−0.06, −0.03)
Low-middle SDI	767,793 (532,015, 1,074,485)	97.38 (66.41, 135.99)	230,461 (157,372, 328,129)	59.55 (40.05, 84.45)	537,331 (367,936, 749,669)	134.04 (91.59,185.74)	1,648,017 (1,137,712, 2,286,803)	96.87 (66.53, 135.34)	575,088 (396,034, 805,313)	66.21 (45.39, 93.2)	1,072,929 (736,877, 1,495,640)	129.02 (88.08, 179.25)	−0.11(−0.15, −0.08)	0.23 (0.19, 0.28)	−0.21 (−0.24, −0.18)
Low SDI	345,556 (236,905, 480,733)	114.32 (78.7, 157.77)	130,462 (89,206, 179,679)	86.31 (59.09, 118.76)	215,094 (147,620, 300,551)	141.76 (98.03,196.54)	791,734 (543,966, 1,102,725)	111.72 (77.54, 154.69)	318,235 (217,911, 440,572)	87.56 (60.26, 120.24)	473,499 (323,863, 664,474)	136.7 (95.04, 190.7)	−0.12 (−0.15, −0.09)	−0.04(−0.11, 0.03)	−0.13(−0.15, −0.11)

#### SDI

In 2021, there were marked variations in the number of YLDs attributed to occupational noise across various SDI regions. The middle SDI region reported the highest number of years YLDs, totaling 3,014,042 person-years (95%UI: 2,027,104–4,241,934). This was followed by the high-middle SDI region, which accounted for 1,654,261 person-years (95%UI: 1,112,237–2,342,966). Conversely, the low SDI region recorded the lowest number of YLDs at 791,734 person-years (95%UI: 543,966–1,102,725). In all five SDI regions, the number of impacted males surpassed that of females (Table 1).

In the SDI quintile regions, the age-standardized YLDs rate saw the most substantial increase in the high-middle SDI region, which rose by 49% (95% UI: 0.47–0.5). Notably, the increase for females was greatly higher at 60.3% (95% UI: 0.61–0.64) compared to 35% (95% UI: 0.33–0.37) for males. The high SDI region exhibited a growth rate of 15% (95% UI: 0.12–0.18), with a notable increase of 33% among females (95% UI: 0.32–0.35), while the male population remained consistent. In the middle SDI, low-middle SDI, and low SDI regions, the overall rates of YLDs, and those specifically for males, have demonstrated a decline. Female YLD rates in the Middle SDI and low-middle SDI regions demonstrated an upward trend, whereas the figures in the low SDI region remained unchanged for females (Table 1).

### Impact of age and gender on the burden of ONIHL

**Figure 1:**
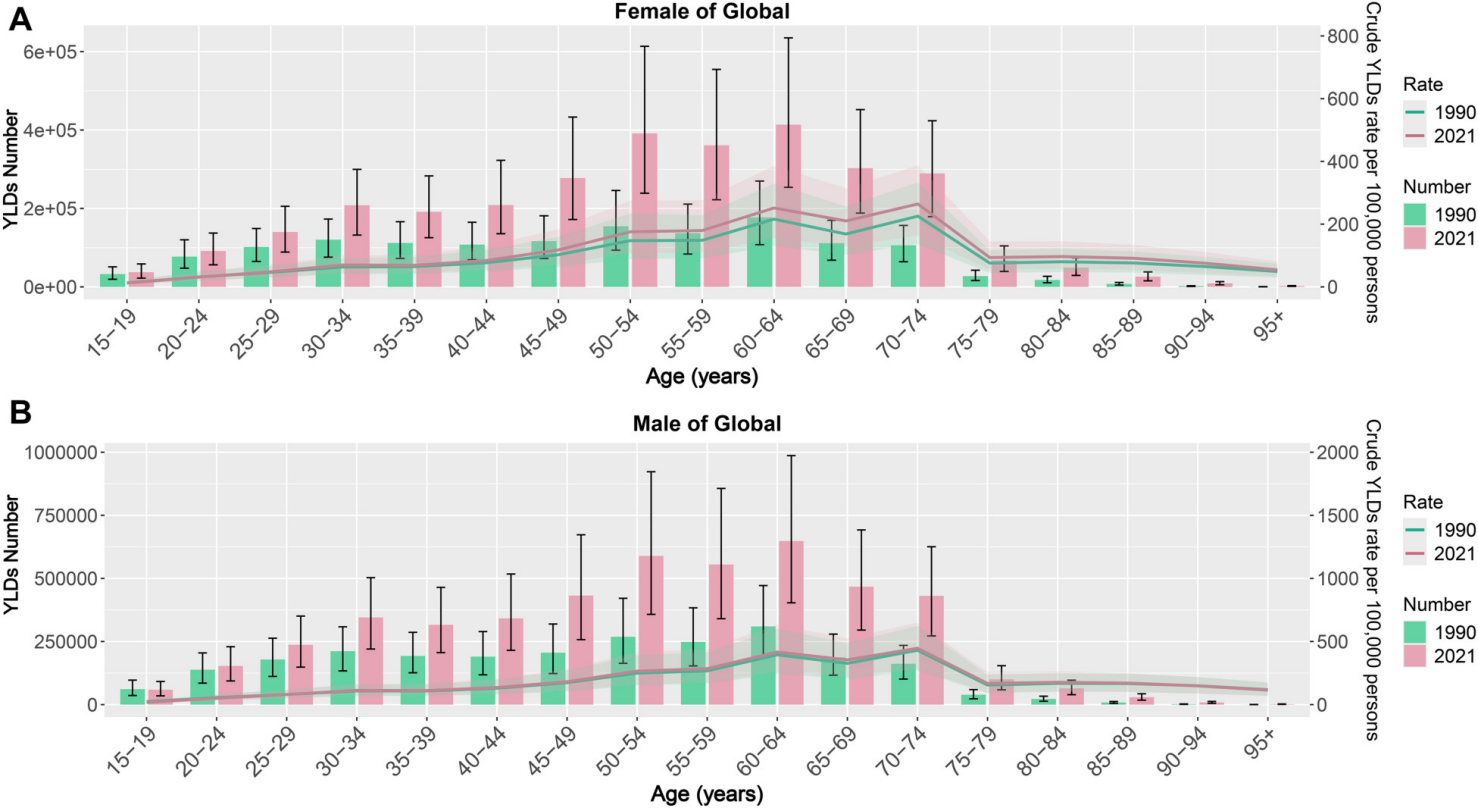
ONIHL burden across different ages and genders [**(A)** Female, **(B)** Male]. ONIHL, Occupational Noise-Induced Hearing Loss.

Both genders experienced an upward trend in the quantity and rate of YLDs associated with noise exposure as age increased, peaking in the 70–74 age group. Across all age categories, male YLDs were consistently higher than those of females (Figure 1). In the five SDI regions, the trends in burden shifts for both males and females across different age groups in high and high-middle SDI areas align with global patterns (Supplementary Figures S1A,B). The number and rate of YLDs are relatively low during middle age but gradually rise in older age. In middle SDI regions, the number and rate of YLDs begin to increase middle a marked escalation observed particularly in those over 50 (Supplementary Figure S1C). Conversely, in low SDI regions, both the number and rate of YLDs start to rise greatly at younger ages (Supplementary Figures S1D,E). What's more, in high-middle SDI regions, the burden for females across various age groups generally exceeds that of males (Supplementary Figure S1B).

### Joinpoint regression analysis of the burden of disease attributable to ONIHL

**Figure 2:**
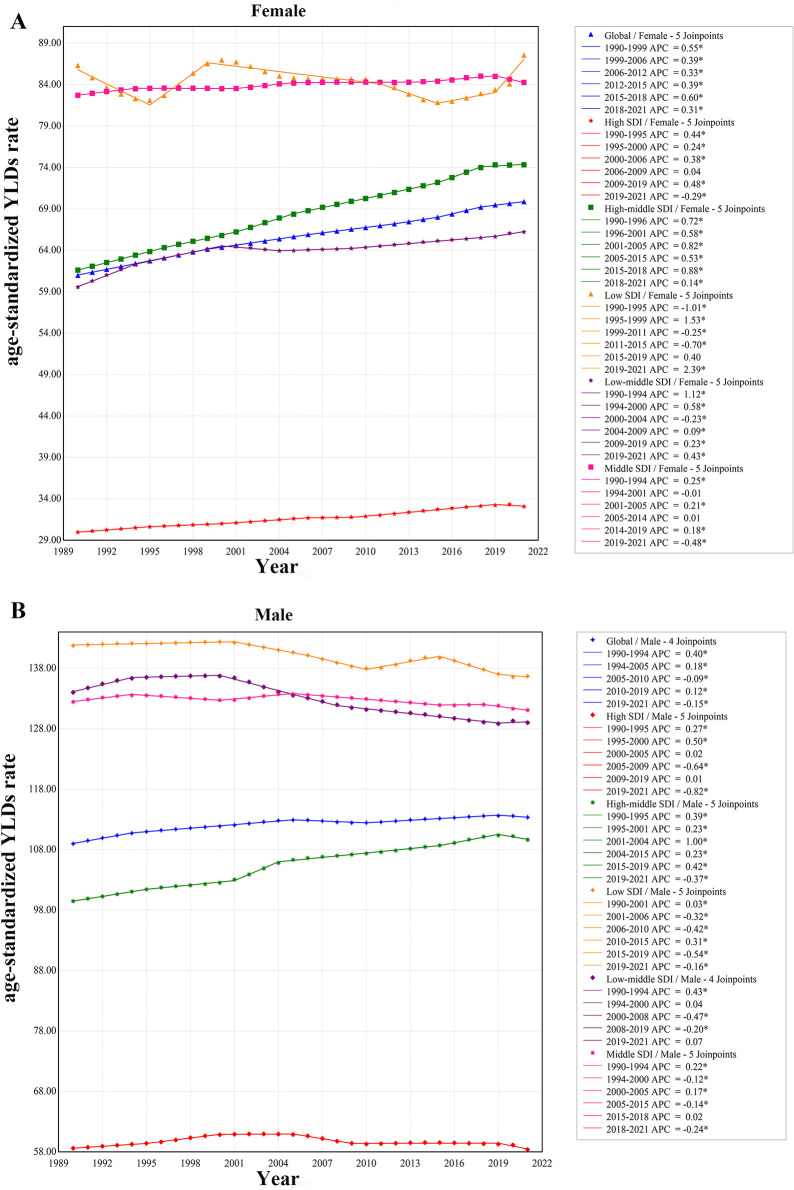
Trends of age-standardized YLDs rate in female **(A)** and male **(B)** of ONIHL in SDI. YLDs, years lived with disability; ONIHL, Occupational Noise-Induced Hearing Loss; SDI, socio-demographic index.

The Joinpoint regression model was employed to analyze the temporal trends of ONIHL burden among different genders globally and across various SDI regions, emphasizing important regional disparities. The global age-standardized YLDs rate for ONIHL exhibited a consistent upward trajectory (APC for 1990–1993: 0.52, 1993–1997: 0.36, 1997–2005: 0.27, 2005–2010: 0.06, 2010–2019: 0.26, 2019–2021: 0.04) (Supplementary Figure S2). The increase observed in females (APC for 1990–1999: 0.55, 1999–2006: 0.39, 2006–2012: 0.33, 2012–2015: 0.39, 2015–2018: 0.60, 2018–2021: 0.31) was markedly more pronounced than that for males (APC for 1990–1994: 0.40, 1994–2005: 0.18, 2005–2010: −0.09, 2010–2019: 0.12, 2019–2021: −0.15), with AAPCs of 0.44 (95% CI: 0.43–0.45) for females and 0.13 (95% CI: 0.12–0.14) for males, respectively (Figure 2; Supplementary Table S2).

In the five SDI regions, the age-standardized YLDs rate for females exhibited an upward trend in the high SDI, high-middle SDI, middle SDI, and low-middle SDI categories. Notably, the high-middle SDI region recorded five important increases (APC from 1990 to 1996 = 0.72, 1996 to 2001 = 0.58, 2001 to 2005 = 0.82, 2005 to 2015 = 0.53, 2015 to 2018 = 0.88, and 2018 to 2021 = 0.14) (Figure 2A). The low-middle SDI region experienced one important decrease and five important increases (APC from 1990 to 1994 = 1.12, 1994 to 2000 = 0.58, 2000 to 2004 = −0.23, 2004 to 2009 = 0.09, 2009 to 2019 = 0.23, and 2019 to 2021 = 0.43) (Figure 2A). The high SDI region also saw five increases (APC from 1990 to 1995 = 0.44, 1995 to 2000 = 0.24, 2000 to 2006 = 0.38, 2006 to 2009 = 0.04, and 2009 to 2019 = 0.48), followed by a decline starting in 2019 (APC from 2019 to 2021 = −0.29) (Figure 2A). The middle SDI region recorded three important increases during the periods 1990–2019 (APC = 0.25), 2001–2005 (APC = 0.21), and 2014–2019 (APC = 0.18), before experiencing an important decrease beginning in 2019 (APC from 2019 to 2021 = −0.48). Conversely, the low SDI region demonstrated no important growth from 1990 to 2021, with an AAPC of 0.05 (95% CI: −0.11 to 0.21) (Supplementary Table S2), however, a marked increase was noted starting in 2019 (APC from 2019 to 2021 = 2.39) (Figure 2A).

In comparison to females, the age-standardized YLDs rate for males across the five SDI regions exhibited a notable increase exclusively in the high-middle SDI region, with an AAPC of 0.32 (95% CI: 0.30, 0.34) (Supplementary Table S2). Conversely, the rates in the middle SDI, low-middle SDI, and low SDI regions demonstrated important decreases, with AAPCs of −0.04, −0.12, and −0.12, respectively (Supplementary Table S2). The decline observed in the high SDI was not statistically important. The high-middle SDI experienced five notable increases and one decrease during the study period, with APCs of 0.39 from 1990 to 1995, 0.23 from 1995 to 2001, 1.00 from 2001 to 2004, 0.23 from 2004 to 2015, 0.42 from 2015 to 2019, and −0.37 from 2019 to 2021 (Figure 2B). While fluctuations in the high SDI over the 32-year span were not statistically important, a marked decline commenced in 2019, with an APC of −0.82 noted for the period from 2019 to 2021 (Figure 2B).

### YLDs rates for 204 countries in 2021 and AAPCs change from 1990 to 2021

**Figure 3:**
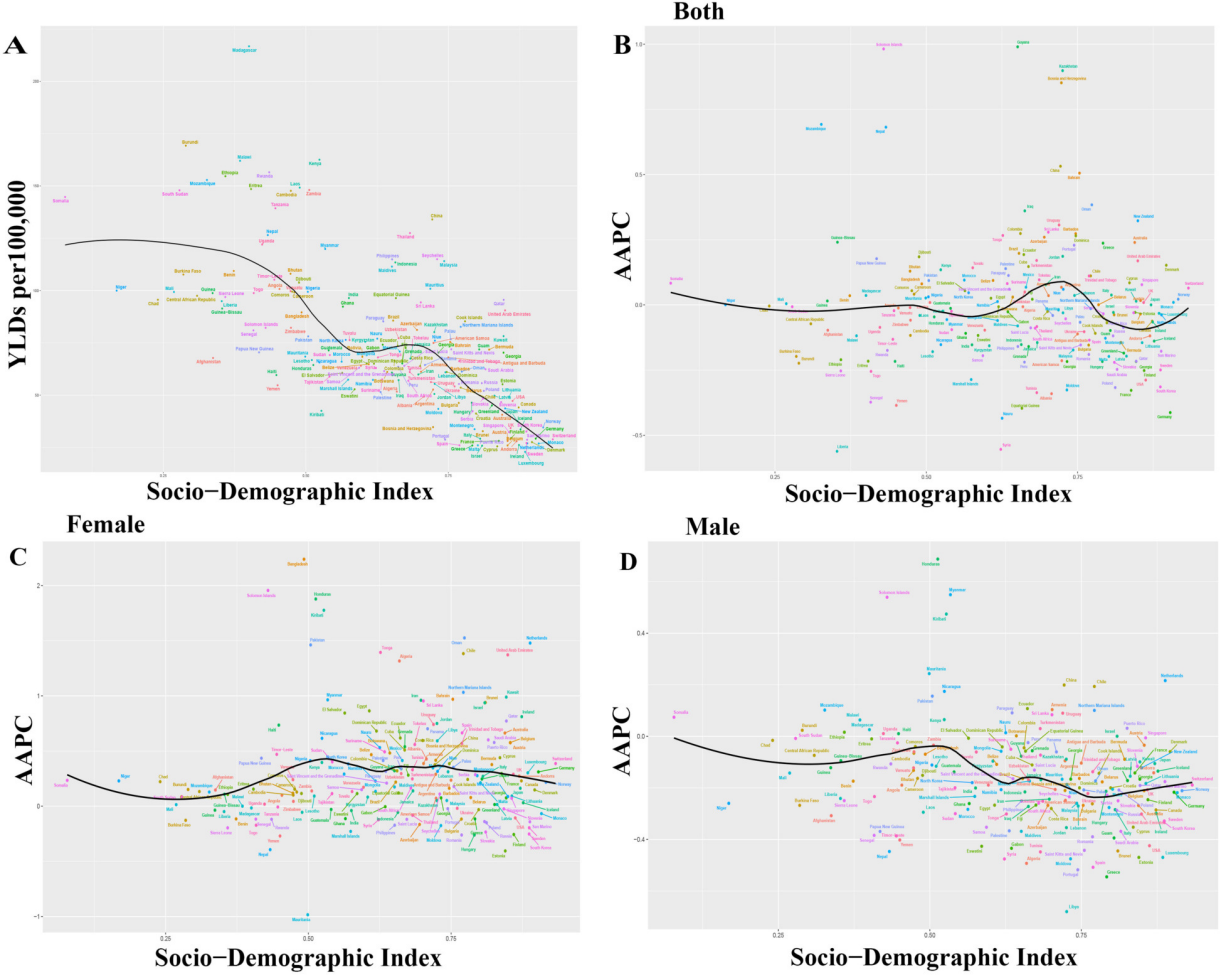
Age-standardized YLDs rate in 204 countries for 2021 and changes in AAPCs from 1990 to 2021. YLDs, years lived with disability; AAPCs, average annual percentage changes. **(A)** Age-standardized YLDs rate for 2021, **(B)** Overall AAPC, **(C)** Female AAPC, **(D)** Male AAPC.

The 2021 data indicates that in the 204 countries, an increase in SDI correlates with a decrease in YLDs rate. The ONIHL burden predominantly occurs within the SDI range of 0.25–0.75, particularly in nations with an SDI between 0.25 and 0.5, where the burden is most pronounced (Figure 3A). Among these, Madagascar experiences the highest burden, reporting 216.77 cases per 100,000 individuals annually, followed by Burundi, Malawi, Rwanda, Ethiopia, and Mozambique. Countries with SDIs between 0.5 and 0.75, such as Kenya, Zambia, China, Thailand, and Myanmar, also endure important burdens (Figure 3A). In nations with an SDI below 0.25, Somalia demonstrates the most acute burden, with 144.698 cases per 100,000 individuals per year, while Qatar shows the highest burden in countries with an SDI exceeding 0.75, at 95.54 cases per 100,000 individuals per year (Figure 3A). This data emphasizes that nations with medium SDI values are most affected by the ONIHL burden.

During the period 1990–2021, several countries experienced a major increase in the AAPC, notably Guyana, the Solomon Islands, Kazakhstan, Bosnia and Herzegovina, Mozambique, Nepal, China, and Bahrain (Figure 3B). In most countries, the AAPC for females showed an upward trend, with Bangladesh exhibiting the most substantial increase at an AAPC of 2.24 (95% CI: 2.21–2.27). This was followed by notable increases in the Solomon Islands, Honduras, Kiribati, Oman, Pakistan, and the Netherlands (Figure 3C). In contrast, the AAPC for males declined in the majority of countries, with notable growth observed in Honduras, Myanmar, the Solomon Islands, Kiribati, Mauritania, the Netherlands, China, and Chile (Figure 3D).

### Analyses of decomposition

**Figure 4:**
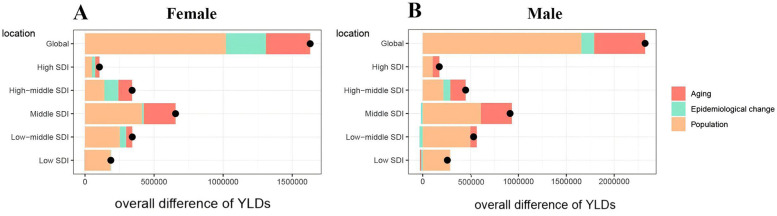
Changes in ONIHL YLDs according to population-level determinants of aging, population growth, and epidemiological change from 1990 to 2021 at the global level and by SDI quintile [**(A)** Female, **(B)** Male]. YLDs, years lived with disability; ONIHL, Occupational Noise-Induced Hearing Loss; SDI, socio-demographic index. The black dot represents the overall value of change contributed by all three components. For each component, the magnitude of a positive value indicates a corresponding increase in YLDs attributed to that component, while the magnitude of a negative value indicates a corresponding decrease in YLDs attributed to the related component.

A decomposition analysis was performed on the original YLDs to assess the individual effects of aging, population growth, and epidemiological changes on ONIHL from 1990 to 2021. The analysis revealed a global upward trend in YLDs for ONIHL across all SDI regions, with the most major increase occurring in middle-SDI regions (Supplementary Figure S3). On a global scale, population growth and aging accounted for 67.81% and 21.34% of the rise in disease burden, respectively (Supplementary Table S3). The effect of population growth was most pronounced in low SDI regions, with an increase of 106.71%, followed by low-middle SDI (86.71%), middle SDI (65.41%), high SDI (54.58%), and high-middle SDI regions (44.73%) (Supplementary Table S3). Aging had a greater impact in higher SDI regions, with contributions of 35.5% in middle SDI, 34.61% in high SDI, 32.16% in high-middle SDI, 13.6% in low-middle SDI, and a negative impact of −3.6% in low SDI regions. In contrast, the contribution of epidemiological changes was relatively minor, with positive effects observed in high SDI (10.8%) and high-middle SDI (23.11%) regions, while negative contributions were noted in middle SDI (−0.91%), low-middle SDI (−0.32%), and low SDI (−3.1%) (Supplementary Table S3). The alterations in male YLD values and the contributions of aging, population growth, and epidemiological factors at the global level and across all SDI regions reflect similar trends. For females, the impacts of population growth and aging are consistent with the global average, while the influence of epidemiological factors is positive (Figures 4A,B).

### Predicted trends of ONIHL YLDs rate in 2022–2036

**Figure 5:**
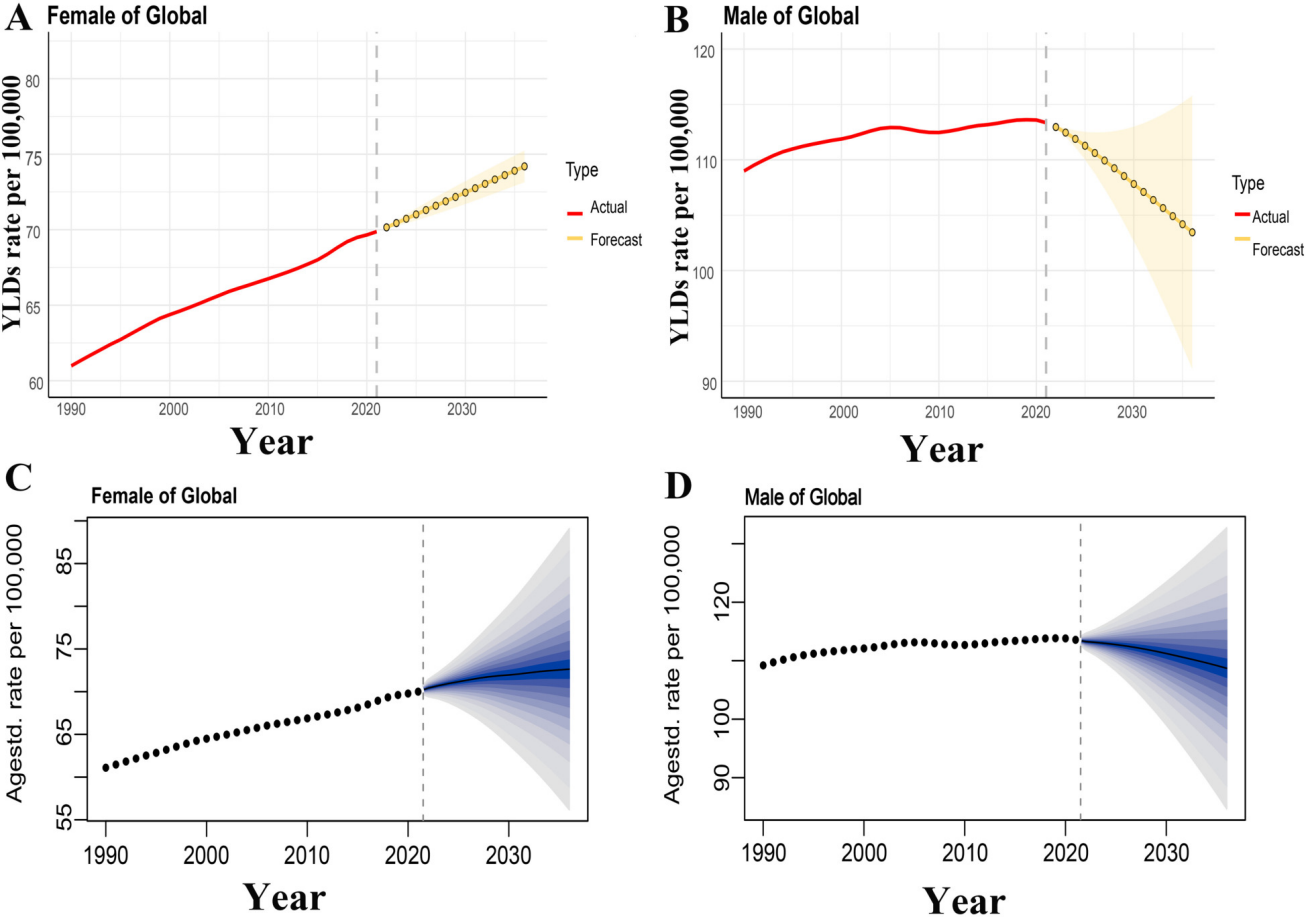
Prediction of ONIHL burden from 2022 to 2036. [**(A,B)** The prediction for males and females based on the ARIMA model. **(C,D)** The prediction for males and females based on the BAPC model. **(A,C)** Age-standardized YLDs rate trend of female from 2022 to 2036 attributable to ONIHL, **(B,D)** age-standardized YLDs rate trend of male from 2022 to 2036 attributable to ONIHL. The yellow lines represent the predicted trend and the light-yellow shaded regions represent the 95% confidence interval of predicted values, the gray dot vertical line split data into true value (1990–2021) and predicted value (2022–2036). YLDs, years lived with disability; ONIHL, Occupational Noise-Induced Hearing Loss; BAPC, Bayesian age-period-cohort; ARIMA, autoregressive integrated moving average.

The ONIHL age-standardized YLDs rate data from 1990 to 2021 was then applied to quantitatively predict future trends over the next fifteen years in ARIMA models and BAPC models. In the ARIMA model, as presented in Supplementary Figures S4A–C, the longitudinal ONIHL YLDs rate were non–stationary, therefore, ﬁrst–order differencing was performed to stabilize the variance of the series (Supplementary Figures S4D–F). The differential time series were further veriﬁed as non–random series through the whitenoise test (Supplementary Table S4). Filtered by the auto.arima() function, the optimized parameters for ARIMA model were chosen to be (1,2,0) for males and (1,1,1) for females, with AICs of −77.39 and −101.75, respectively. Q–Q plots, ACF and PACF plots revealed that the residual error was normally distributed (Supplementary Figure S6). The Ljung–Box test conﬁrmed that ARIMA models were robust and the residuals were white noise (*χ*^2^ = 7.421/13.28, *p* = 0.826/0.349). ARIMA (1,2,0) and ARIMA (1,1,1) models were then used to predict YLDs rate of ONIHL from 2022 to 2036 by gender, as displayed in Figure 5. YLDs rate in males is expected to decrease from 113.35 per 100,000 in 2021 to 103.45 per 100,000 in 2036, whereas YLDs rate in females will Continuously rise in the next fifteen years, increasing from 69.87 per 100,000 in 2021 to 74.19 per 100,000 in 2036. The predictive capacity of ARIMA models was listed in Supplementary Table S5. Figures 5C,D illustrates the BAPC model predictions for YLDs rate for both males and females from 2022 to 2036, with the predicted trends being similar to those of the ARIMA model.

## Discussion

Occupational noise exposure is a widespread issue affecting worker health around the world. Workers who spend long hours in noisy environments not only risk hearing loss but also face a range of other health problems, including cardiovascular diseases, sleep disorders, and stress-related issues (22). These challenges not only diminish the quality of life for workers but also contribute to an increase in YLDs, placing a heavy burden on society's economy. That's why it's essential to study the impact of occupational noise exposure on YLDs. This research specifically aims to explore how noise exposure affects YLDs, with a focus on the growing numbers among females and the trends we foresee in the future. We'll discuss the potential causes and mechanisms behind hearing loss in female to offer targeted insights and recommendations for addressing these concerns effectively.

### Shifts in occupational structures are increasing opportunities for female

Our research emphasizes an important increase in age-standardized YLDs rates in the middle SDI regions, particularly among female. These areas are currently undergoing a transformation in their economic structures as they industrialize. While men still make up the majority of the workforce, there's a noticeable rise in the number of Women entering traditionally male-dominated sectors, like manufacturing and construction, which are often associated with high levels of noise pollution (23). This shift in job roles is contributing to an increased cumulative risk of occupational noise exposure for female. On top of that, the medical resources in these middle SDI regions are relatively sufficient, and there's a wider availability of hearing screenings. This means that cases are being diagnosed and reported more accurately, finally leading to a higher detection rate of hearing-related issues.

Female in high-to-middle SDI regions may be more likely to work in service industries such as healthcare, education, and catering. While these jobs aren't usually labeled as “high-noise occupations,” prolonged exposure to background sounds—like equipment noise and the hustle and bustle of crowds—can still lead to cumulative hearing damage. This finding is corroborated by the literature data referenced later. For instance, research indicates that 16.0% of female orthopedic surgeons have experienced some degree of hearing loss since starting their careers, with 5.3% officially diagnosed with noise-induced hearing loss (24). In intensive care units (ICUs), where the constant activity of medical staff, combined with the sounds of alarms from monitoring equipment, creates sound levels that often surpass national and international guidelines (25). Similarly, a Swedish study focused on women preschool teachers showed that they experience higher rates of hearing loss, tinnitus, and sensitivity to sound compared to a control group. In fact, preschool teachers are at a greater risk for hearing-related issues than women in the general population (26).

In regions with low SDI, the YLDs rate is noticeably higher compared to areas with high SDI. This trend can be attributed to several factors. Female in low SDI regions often work in informal sectors where monitoring of noise exposure is lacking due to limited healthcare resources. Also, these areas tend to rely heavily on noisy industries and have inadequate labor protection systems, which likely contributes to the enhanced disability rates.

### Impact of changes in social demographics

In 2021, there was a notable increase in YLDs attributable to occupational noise compared to 1990, primarily influenced by population growth and aging. Gender disparities reveal that the global burden of ONIHL is greatly higher in male than in female, corroborating earlier research findings (21). Occupational noise exposure is particularly common in sectors such as mining, manufacturing, and construction, which typically employ a larger share of male workers, thus intensifying the noise exposure burden for men (9). However, the situation is changing as more women are entering the workforce. According to the 2024 Global Gender Gap report, more and more women are entering the job market, particularly in entry-level positions, which now accounts for almost half of the workforce (27). This growth means that a larger segment of the population is exposed to occupational noise. Another factor to consider is the aging workforce and the cumulative effects of exposure over time. Prolonged noise exposure can have long-term negative impacts on hearing (28). Our research also indicates that YLDs among female peak at ages 70–74. This could be linked to women's longer life expectancy and the decreased protective effects of estrogen (29). Looking at it from a different angle, the long-term effects of chronic occupational exposure might be intensified due to women's tendency to live longer.

### Interaction between biological and social factors

Noise can have a major impact on the protein levels in our cochlea, which in turn affects our hearing (30). Research indicates that estrogen activates the ERα/ERβreceptors in the cochlea's stria vascularis, helping to regulate oxidative stress pathways and counteract hearing issues caused by noise exposure (29). This might be one reason why people have historically believed that female are less affected by noise than men (31). Studies show that estrogen has a direct effect on auditory nerve function and blood flow in the cochlea. A decline in estrogen levels is closely linked to an early increase in high-frequency hearing loss, suggesting that postmenopausal women are at a greater risk for hearing impairment (32). In our research, we noted that the rate of YLDs for female tends to rise sharply with age, peaking around 70–74 years. This increasing likely correlates with the major drop in estrogen levels during menopause. However, the current occupational health assessment systems haven't yet taken into account female's hormonal statuses, like screening for perimenopausal changes. This oversight means that the strategies we have in place to protect female's hearing often lack a strong biological foundation.

Research indicates that when noise levels reach 83 decibels, women are more susceptible to hearing loss than men, particularly at high frequencies (33). Certain noisy occupations set permissible exposure limits at 85 decibels, and in such environments, workers may neglect to use hearing protection, thus increasing their risk of noise exposure (8). Research has shown that race plays a role in hearing health. A study found that Black women face a higher risk of hearing loss compared to other racial and gender groups, with an odds ratio of 2.9 (34). Besides, both Black and Brown women, particularly those with lower socioeconomic status, may find themselves at a disadvantage when it comes to accessing hearing healthcare. This can result in them recognizing their hearing loss only when it has progressed greatly (29).

### Dual impact of work hours and noise exposure intensity

Noise-induced hearing loss can be greatly affected by two main factors: the duration of exposure at work and the intensity of the noise itself. Industries like textiles, which employ primarily women, have particularly high levels of noise exposure (35, 36). For instance, a study emphasized that Surakarta, one of India's largest textile companies, struggles with noise levels that exceed the Threshold Limit Value. This issue has led to various health concerns, such as enhanced blood pressure, irregular pulse rates, and hearing loss (37). What's more, it's important to note that even if noise levels are within safe limits, factors like heavy workloads and long hours can still heighten the risk of cumulative damage to hearing over time (35, 36).

### Differences in hearing protection devices use between genders

Wearing hearing protection devices (HPDs) can greatly reduce the risk of hearing damage caused by noise, making sound attenuation an essential step in preventing hearing loss (38). Even occasional users of these devices enjoy better protection compared to those who have never used them (39). However, research has shown that women tend to use hearing protection equipment less frequently than men (40). For example, a survey in India's textile industry found that only 29% of workers recognized the potential dangers of noise to their health, and fewer than 30% reported wearing earplugs (41). In the U.S, about 50.7% of women exposed to noise use HPDs, compared to 68.9% of men (23). Also, a report from the European Union on women's work conditions emphasized that women generally receive less training and guidance on HPDs usage than men, which could be a contributing factor to their lower usage rates (23). Comfort and fit are also important issues, existing HPDs, like earplugs and earmuffs, may not always be suitable for female's ear anatomy, which can affect their willingness to wear them (38).

### Upcoming challenges in addressing health risks related to female's noise exposure

In the forecast for the next 15 years, the YLDs rate for males is projected to show a declining trend, with the following main reasons considered, (1). Upgraded protection in traditional high-noise industries: Industries such as manufacturing and construction, which are predominantly male-dominated and traditionally associated with high noise levels, have seen improvements in noise control in recent years due to technological advancements and policy changes (42). (2). Personal protective measures: Implementing personal protective measures is crucial for preventing occupational diseases, particularly by providing appropriate hearing protection to individuals exposed to excessive noise. This approach is now widely adopted by enterprises (43). (3). Institutionalization of occupational health monitoring: The government has established relevant laws and regulations, while enterprises have created occupational health records, conducted regular hearing screenings, enabling the early detection of hearing damage and early intervention.

However, In the 15-year forecast, the YLDs rate for females is predicted to rise instead of decline. This projected outcome is likely driven by a combination of factors, including the changing gender-differentiated occupational structure, disparities in industry protection measures, and physiological factors. Building upon the previous discussion, the specific reasons considered are as follows: 1. Occupational Factors: (a) An increasing participation rate of women in the labor market (27); (b) A significant growth trend in the occupational penetration of women into traditionally male-dominated noise-intensive industries (23); (c) “Feminization” of emerging noise-exposure industries: Service sectors with a high proportion of female workers (e.g, education, healthcare) may contain new sources of noise exposure (25, 26), but these are often not included in routine regulations, leading to low awareness and long-term neglect of protective measures (35, 36); (d) For female-dominated high-noise-exposure industries, such as the textile industry, even if noise exposure levels do not exceed safety thresholds, high-intensity workloads and long overtime hours may still increase the risk of cumulative damage. 2. Gender Factors: (a) Women have a longer life expectancy, and the lack of estrogen protection after menopause may lead to cumulative exposure effects (29); (b) Gender differences in the use of hearing protection devices have not been adequately recognized; (c) When noise levels reach 83 decibels, women are more susceptible to hearing loss than men, particularly in the high-frequency range (33); (d) Dual exposure burden: In addition to occupational noise, women often bear a greater burden of domestic noise exposure (e.g, from household appliances), creating a cycle of “all-day noise exposure damage”. 3. Other Factor: Compared to other gender/ethnic groups, the risk associated with hearing loss appears to be stronger in Black women (34).

Even though more females are entering the workforce, the current occupational health and safety systems still have major gaps when it comes to gender considerations. Many industries are still applying outdated standards that don't adequately address the unique health risks female face—like the recognition of occupational injuries, protective gear, and health management strategies. One striking trend is that the increase in noise-related YLDs among female is surpassing that of male globally. If this trend continues, we can expect to see a notable rise in YLDs for female due to noise-related issues over the next 15 years. This is a reminder that we need to pay more attention to hearing loss in female. For instance, when setting standards for occupational noise levels, it's critical to take gender differences into account. Some non-traditional noisy environments, such as ICUs, agriculture, healthcare, and education, have a higher percentage of female workers, so we should consider how these conditions affect their hearing. Besides, we should design HPDs that cater specifically to female and include female figures in our awareness campaigns to help them find role models for using these devices. Finally, it's essential to enhance government oversight to raise awareness among females about the dangers of noise exposure, and to provide them with training and guidance on the proper use of hearing protection equipment.

### Burden of ONIHL varies among different countries

According to data from 2021 spanning 204 countries, an increase in the SDI corresponds with a general decline in the age-standardized YLDs rates. Over the past 32 years, the AAPC for males has predominantly decreased across most countries, while females have displayed an opposing trend, aligning with previous findings. In 2021, the burden of ONIHL was primarily concentrated in nations with an SDI value between 0.25 and 0.75, with countries in the medium SDI range experiencing the greatest impact. This trend can be attributed to their large, rapidly expanding populations and their position in the mid-industrialization phase, where reliance on manufacturing and related industries for economic growth has outpaced the implementation of adequate occupational health protections. Besides, the simplicity of hearing diagnostic technology has contributed to a rise in reported cases of hearing loss. Among the 204 countries assessed, Madagascar exhibited the highest burden of ONIHL, followed closely by Burundi, Malawi, and Rwanda—all located in Africa—where burgeoning populations and an important proportion of mining and informal economic activities may exacerbate the disease burden (44). In regions with an SDI below 0.25, Somalia stands out with the most acute ONIHL burden, attributable to factors such as prolonged instability, inadequate infrastructure, weak regulatory frameworks, a high disease burden, and potential underreporting. In contrast, nations with an SDI exceeding 0.75, such as Qatar, face important ONIHL challenges, likely linked to their rich oil and natural gas resources, swift urbanization, population increase, extensive construction activities, and a surge in automobile usage (45).

### Occupational noise exposure in low SDI regions: hidden concern of younger-onset hearing loss

The number and rate of YLDs resulting from occupational noise-induced hearing loss increase with age, reaching their highest point in the 70–74 age demographic, as supported by existing research (9). However, in areas with low and medium-low SDI, there is a notable trend toward younger individuals being affected by noise-related damage. This shift can be attributed to several factors: Firstly, economies in these regions often depend heavily on high-noise industries such as manufacturing, construction, and mining, which necessitate considerable physical labor and employ younger workers. Besides, familial economic pressures and lower educational attainment lead to early workforce entry for young people, who are exposed to hazardous noise levels from a young age. This is particularly evident in informal industries where basic protective equipment is often lacking. For instance, at a small-scale gold mine in Tarkwa, worker ages range from 17 to 72, with 70% of employees lacking any protective measures (46). Secondly, regulatory enforcement and oversight in these regions are often insufficient. While certain countries have established limits for occupational noise exposure, implementation is frequently weak, and compliance from companies is minimal. There is also a general lack of public awareness regarding occupational health issues, which hampers the effectiveness of protective measures. Even when earplugs and earmuffs are provided, workers may not fully comprehend the risks associated with noise exposure or the correct usage of protective gear, rendering these safety measures ineffective. Thirdly, noise exposure initially affects higher frequencies, which do not greatly hinder everyday communication. The limited availability of medical resources, coupled with a lack of awareness regarding occupational hazards, often leads to the inability to detect early signs of hearing damage among young workers in a timely manner. This results in cumulative damage, contributing to the trend of increased risk among younger age groups. Fourthly, compared to high SDI regions and countries, low SDI regions may have relatively inadequate access to healthcare services. Workers in these areas may find it difficult to detect hearing loss early, and even when experiencing symptoms such as hearing decline or tinnitus, they may not receive timely medical care, which can easily lead to accumulated exposure.

## Limitation

This study acknowledges several limitations. Firstly, GBD data is dependent on the reporting systems of various regions, which may contribute to the underreporting of certain occupational noise epidemiological data, particularly in areas with medium to low SDI. Secondly, the constraints of the GBD data allow for only a general assessment of the burden of ONIHL, without an in-depth analysis of its impact on employees across different sectors, even though this burden can differ considerably among various industries (47). Lastly, due to insufficient data, we were unable to classify occupational noise into distinct categories, such as steady-state and non-steady-state noise, preventing us from accurately estimating the hearing loss burden caused by different types of noise exposure.

## Conclusion and recommendation

Occupational noise exposure isn't just a workplace health issue, it's a global challenge that has serious implications for social and economic development. The rising burden of ONIHL among females reflects major changes in the global labor market, and shortcomings in our occupational health systems. Moving forward, we need more research to discover how biological, social, and policy factors intersect. It's also important to include a gender perspective when developing occupational health standards to ensure fair prevention and control of hearing loss. Besides, we should tackle noise control from various angles—like technology, policy, and culture—to create a more effective protective framework for all workers, finally helping to lessen the burden of related diseases.

## Data Availability

The datasets presented in this study can be found in online repositories. The names of the repository/repositories and accession number(s) can be found below: website of IHME (http://ghdx.healthdata.org/).

## References

[1] Age-related and other hearing loss - Level 3 cause | Institute for Health Metrics and Evaluation. Available online at: https://www.healthdata.org/research-analysis/diseases-injuries-risks/factsheets/2021-age-related-and-other-hearing-loss-level (Accessed February 18, 2025).

[2] Chen K-H Su S-B Chen K-T. An overview of occupational noise-induced hearing loss among workers: epidemiology, pathogenesis, and preventive measures. Environ Health Prev Med. (2020) 25(1):65. 10.1186/s12199-020-00906-0 33129267 PMC7603754

[3] Masterson EA Bushnell PT Themann CL Morata TC. Hearing impairment among noise-exposed workers - United States, 2003–2012. MMWR Morb Mortal Wkly Rep. (2016) 65(15):389–94. 10.15585/mmwr.mm6515a2 27101435

[4] Lusk SL Gillespie B Hagerty BM Ziemba RA. Acute effects of noise on blood pressure and heart rate. Arch Environ Health. (2004) 59(8):392–9. 10.3200/AEOH.59.8.392-399 16268115

[5] Sayler SK Rabinowitz PM Cantley LF Galusha D Neitzel RL. Costs and effectiveness of hearing conservation programs at 14 US metal manufacturing facilities. Int J Audiol. (2018) 57(sup1):S3–S11. 10.1080/14992027.2017.1410237 29216778 PMC6188788

[6] Selander J Albin M Rosenhall U Rylander L Lewné M Gustavsson P. Maternal occupational exposure to noise during pregnancy and hearing dysfunction in children: a nationwide prospective cohort study in Sweden. Environ Health Perspect. (2016) 124(6):855–60. 10.1289/ehp.1509874 26649754 PMC4892921

[7] Wang Q Wang X Yang L Han K Huang Z Wu H. Sex differences in noise-induced hearing loss: a cross-sectional study in China. Biol Sex Differ. (2021) 12(1):24. 10.1186/s13293-021-00369-0 33676563 PMC7937304

[8] Eng A Mannetje A McLean D Ellison-Loschmann L Cheng S Pearce N. Gender differences in occupational exposure patterns. Occup Environ Med. (2011) 68(12):888–94. 10.1136/oem.2010.064097 21486991

[9] Wang S Liu S Li K Tang W Fan X Cheng Y A systematic analysis of the burden of disease attributable to occupational noise-induced hearing loss in China based on the 2019 global burden of disease study. BMC Public Health. (2024) 24(1):3423. 10.1186/s12889-024-21094-4 39695537 PMC11654318

[10] Vos T Flaxman AD Naghavi M Lozano R Michaud C Ezzati M Years lived with disability (YLDs) for 1160 sequelae of 289 diseases and injuries 1990–2010: a systematic analysis for the global burden of disease study 2010. Lancet Lond Engl. (2012) 380(9859):2163–96. 10.1016/S0140-6736(12)61729-2 PMC6350784 23245607

[11] Deafness and hearing loss. Available online at: https://www.who.int/news-room/fact-sheets/detail/deafness-and-hearing-loss (Accessed February 18, 2025).

[12] GBD 2019 Hearing Loss Collaborators. Hearing loss prevalence and years lived with disability, 1990–2019: findings from the global burden of disease study 2019. Lancet Lond Engl. (2021) 397:996–1009. 10.1016/S0140-6736(21)00516-X PMC7960691 33714390

[13] Occupational noise - Level 3 risk | Institute for Health Metrics and Evaluation. Available online at: https://www.healthdata.org/research-analysis/diseases-injuries-risks/factsheets/2021-occupational-noise-level-3-risk (Accessed February 18, 2025).

[14] Global Burden of Disease Study 2021 (GBD 2021) Socio-Demographic Index (SDI) 1950–2021 | GHDx. Available online at: https://ghdx.healthdata.org/record/global-burden-disease-study-2021-gbd-2021-socio-demographic-index-sdi-1950%E2%80%932021 (Accessed January 11, 2025).

[15] Kim HJ Fay MP Feuer EJ Midthune DN. Permutation tests for joinpoint regression with applications to cancer rates. Stat Med. (2000) 19(3):335–51. 10.1002/(sici)1097-0258(20000215)19:3<335::aid-sim336>3.0.co;2-z 10649300

[16] Das Gupta P. Standardization and decomposition of rates from cross-classified data. Genus. (1994) 50:171–96. 12319256

[17] Nguyen HV Naeem MA Wichitaksorn N Pears R. A smart system for short-term price prediction using time series models. Comput Electr Eng. (2019) 76:339–52. 10.1016/j.compeleceng.2019.04.013

[18] Nazari KangavariHShojaeiAHashemi NazariSS. Suicide mortality trends in four provinces of Iran with the highest mortality, from 2006 to 2016. J Res Health Sci. (2017) 17:e00382.28676594

[19] Li Y Ning Y Shen B Shi Y Song N Fang Y Temporal trends in prevalence and mortality for chronic kidney disease in China from 1990 to 2019: an analysis of the global burden of disease study 2019. Clin Kidney J. (2023) 16:312–21. 10.1093/ckj/sfac218 36755850 PMC9900593

[20] Wu B Li Y Shi B Zhang X Lai Y Cui F Temporal trends of breast cancer burden in the Western Pacific region from 1990 to 2044: implications from the global burden of disease study 2019. J Adv Res. (2024) 59:189–99. 10.1016/j.jare.2023.07.003 37422280 PMC11082062

[21] Knoll M Furkel J Debus J Abdollahi A Karch A Stock C. An R package for an integrated evaluation of statistical approaches to cancer incidence projection. BMC Med Res Methodol. (2020) 20(1):257. 10.1186/s12874-020-01133-5 33059585 PMC7559591

[22] Morata TC Gong W Tikka C Samelli AG Verbeek JH. Hearing protection field attenuation estimation systems and associated training for reducing workers’ exposure to noise. Cochrane Database Syst Rev. (2024) 5(5):CD015066. 10.1002/14651858.CD015066.pub2 38757544 PMC11099959

[23] Meira TC Santana VS Ferrite S. Gender and other factors associated with the use of hearing protection devices at work. Rev Saude Publica. (2015) 49:76. S0034-89102015000100259. 10.1590/S0034-8910.2015049005708 26487294 PMC4603261

[24] Sedani AB Yakkanti RR Syros A Swonger RM LaPorte DM Aiyer AA An overview of occupational injuries among female orthopaedic surgeons. J Orthop. (2024) 47:94–9. 10.1016/j.jor.2023.10.037 38046449 PMC10686843

[25] Tahvili A Waite A Hampton T Welters I Lee PJ. Noise and sound in the intensive care unit: a cohort study. Sci Rep. (2025) 15:10858. 10.1038/s41598-025-94365-8 40157982 PMC11955003

[26] Fredriksson S Kim J-L Torén K Magnusson L Kähäri K Söderberg M Working in preschool increases the risk of hearing-related symptoms: a cohort study among Swedish women. Int Arch Occup Environ Health. (2019) 92:1179–90. 10.1007/s00420-019-01453-0 31286224 PMC6814644

[27] Global Gender Gap Report 2024. World Econ Forum. Available online at: https://www.weforum.org/publications/global-gender-gap-report-2024/digest/ (Accessed April 14, 2025).

[28] Dillard LK Humes LE Matthews LJ Dubno JR. Noise exposure history and age-related changes to hearing. JAMA Otolaryngol Head Neck Surg. (2025) 151:228–35. 10.1001/jamaoto.2024.4768 39786765 PMC11907306

[29] Reavis KM Bisgaard N Canlon B Dubno JR Frisina RD Hertzano R Sex-linked biology and gender-related research is essential to advancing hearing health. Ear Hear. (2023) 44:10–27. 10.1097/AUD.0000000000001291 36384870 PMC10234332

[30] Jain RK Pingle SK Tumane RG Thakkar LR Jawade AA Barapatre A Cochlear proteins associated with noise-induced hearing loss: an update. Indian J Occup Environ Med. (2018) 22:60–73. 10.4103/ijoem.IJOEM_43_18 30319226 PMC6176698

[31] Delhez A Lefebvre P Péqueux C Malgrange B Delacroix L. Auditory function and dysfunction: estrogen makes a difference. Cell Mol Life Sci CMLS. (2020) 77(4):619–35. 10.1007/s00018-019-03295-y 31522250 PMC11105012

[32] Trott S Cline T Weihing J Beshear D Bush M Shinn J. Hormones and hearing: central auditory processing in women. J Am Acad Audiol. (2019) 30(6):493–501. 10.3766/jaaa.17123 30461407

[33] Lusk SL Ronis DL Baer LM. Gender differences in blue collar workers’ use of hearing protection. Women Health. (1997) 25(4):69–89. 10.1300/J013v25n04_04 9302730

[34] Helzner EP Cauley JA Pratt SR Wisniewski SR Zmuda JM Talbott EO Race and sex differences in age-related hearing loss: the health, aging and body composition study. J Am Geriatr Soc. (2005) 53:2119–27. 10.1111/j.1532-5415.2005.00525.x 16398896

[35] Fu S Huang L Zhong X Su S Li X Huang Q Association between personal attenuation rating with types of earplugs and noise exposure levels in a textile factory in China. Int J Audiol. (2024) 10:1–7. 10.1080/14992027.2024.2396028 39387323

[36] Nguyen AL Nguyen TC Van TL Hoang MH Nguyen S Jonai H Noise levels and hearing ability of female workers in a textile factory in Vietnam. Ind Health. (1998) 36(1):61–5. 10.2486/indhealth.36.61 9473860

[37] Chahyadhi B Widjanarti MP Ada’ YR Suratna FSN Wijayanti R. Noise intensity, blood pressure, and pulse rate in textile industry workers. Placentum J Ilm Kesehat Dan Apl. (2022) 10:73. 10.20961/placentum.v10i1.53146

[38] Kwak C Han W. The effectiveness of hearing protection devices: a systematic review and meta-analysis. Int J Environ Res Public Health. (2021) 18:11693. 10.3390/ijerph182111693 34770206 PMC8583416

[39] Bandyopadhyay A Mukherjee A Dhar G Rout AJ. Burden and risk factors of occupational noise-induced hearing loss among employees working at an international airport in eastern India. Indian J Occup Environ Med. (2024) 28(3):223–7. 10.4103/ijoem.ijoem_163_23 39618895 PMC11606559

[40] McCullagh MC Banerjee T Yang JJ Bernick J Duffy S Redman R. Gender differences in use of hearing protection devices among farm operators. Noise Health. (2016) 18(85):368–75. 10.4103/1463-1741.195803 27991469 PMC5227018

[41] Bedi R. Evaluation of occupational environment in two textile plants in northern India with specific reference to noise. Ind Health. (2006) 44(1):112–6. 10.2486/indhealth.44.112 16610545

[42] Lewkowski K Heyworth JS McCausland K Williams W Fritschi L. Sources of noise exposure across Australian workplaces: cross-sectional analysis and modelling the impact of a targeted noise-source reduction initiative. Ann Work Expo Health. (2024) 68(6):626–35. 10.1093/annweh/wxae029 38795381

[43] Zhang C Wang J Wang H Zhang H. Surveillance of noise exposure level in industrial enterprises-Jiangsu province, China, 2022. Front Public Health. (2024) 12:1230481. 10.3389/fpubh.2024.1230481 38410664 PMC10894969

[44] Khoza-Shangase K Moroe NF Edwards A. Occupational hearing loss in Africa: an interdisciplinary view of the current status. South Afr J Commun Disord. (2020) 67(2):e1–3. 10.4102/sajcd.v67i2.700 PMC7136810 32129656

[45] Abdur-Rouf K Shaaban K. Measuring, mapping, and evaluating daytime traffic noise levels at urban road intersections in Doha, Qatar. Future Transp. (2022) 2:625–43. 10.3390/futuretransp2030034

[46] Calys-Tagoe BNL Ovadje L Clarke E Basu N Robins T. Injury profiles associated with artisanal and small-scale gold mining in Tarkwa, Ghana. Int J Environ Res Public Health. (2015) 12(7):7922–37. 10.3390/ijerph120707922 26184264 PMC4515700

[47] Sayler SK Roberts BJ Manning MA Sun K Neitzel RL. Patterns and trends in OSHA occupational noise exposure measurements from 1979 to 2013. Occup Environ Med. (2019) 76(2):118–24. 10.1136/oemed-2018-105041 30482879 PMC9928427

