# Supplementary material for: Global impact of Occupational Noise-Induced Hearing Loss (ONIHL): trends, gender disparities, and future projections: 1990–2036
Source: Front Glob Womens Health. 2025 Jul 22;6:1584639. doi: 10.3389/fgwh.2025.1584639 (PMC12321882; doi:10.3389/fgwh.2025.1584639)
Supplement: Supplementary file 1 [file Datasheet1.docx]

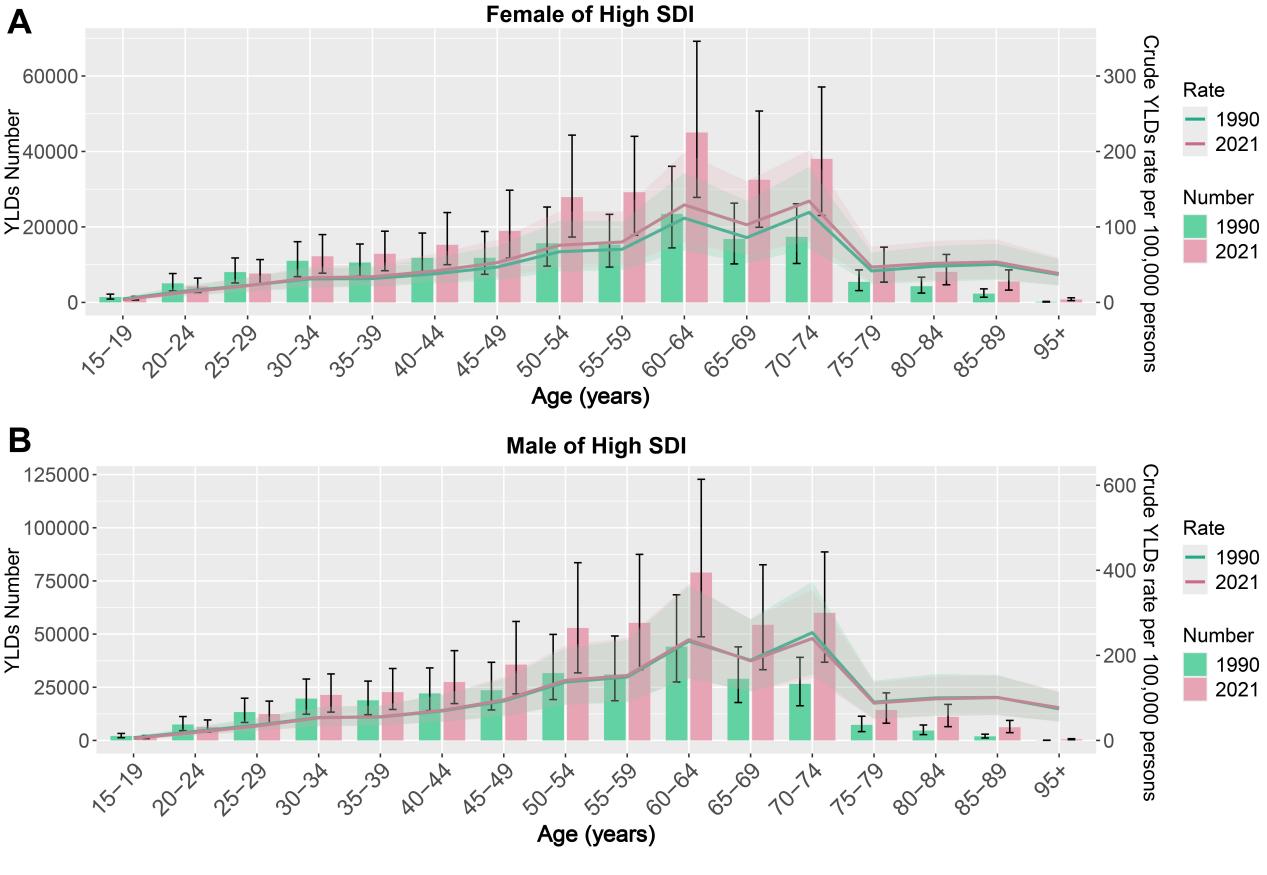


**Supplementary Figure S1(1). Burden of ONIHL among different genders and ages in High SDI region**


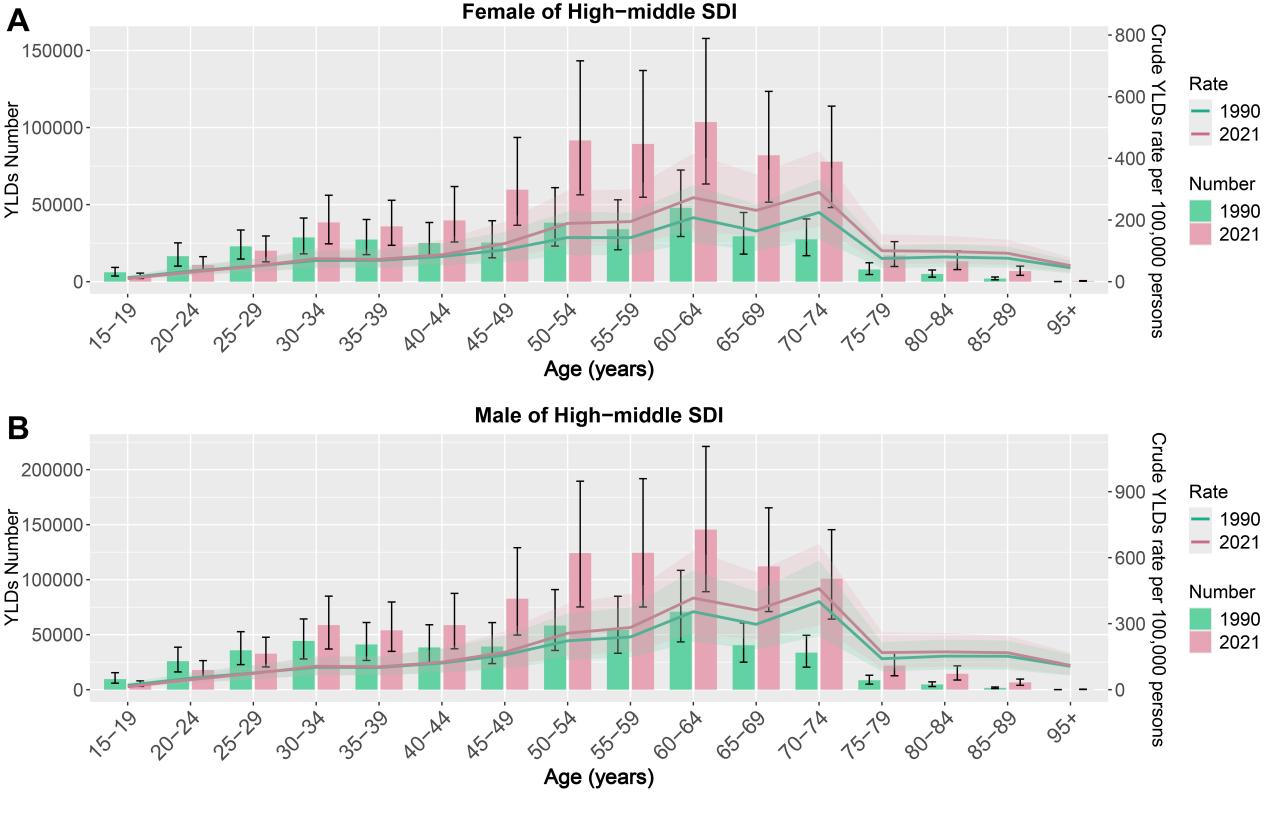


**Supplementary Figure S1(2). Burden of ONIHL among different genders and ages in High-middle SDI region**

**
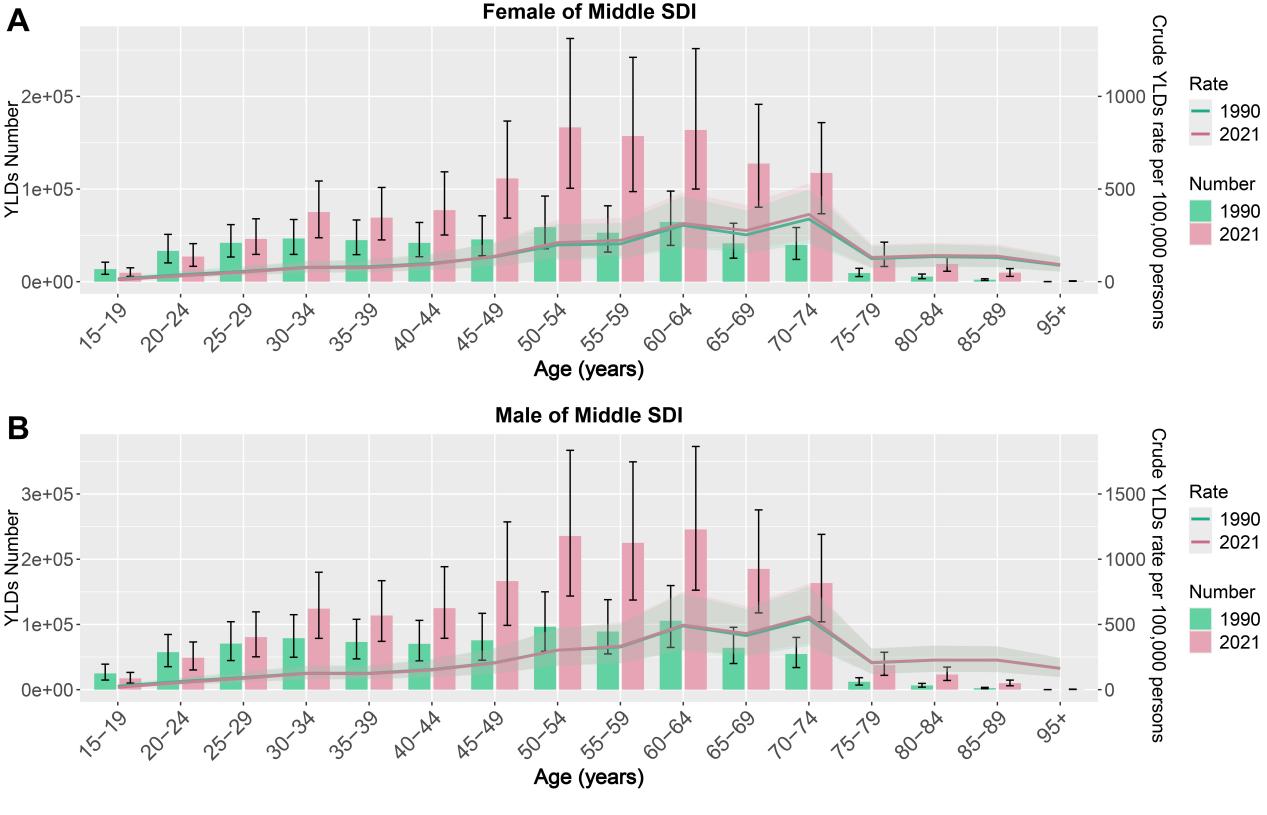
**

**Supplementary Figure S1(3). Burden of ONIHL among different genders and ages in Middle SDI region**

**
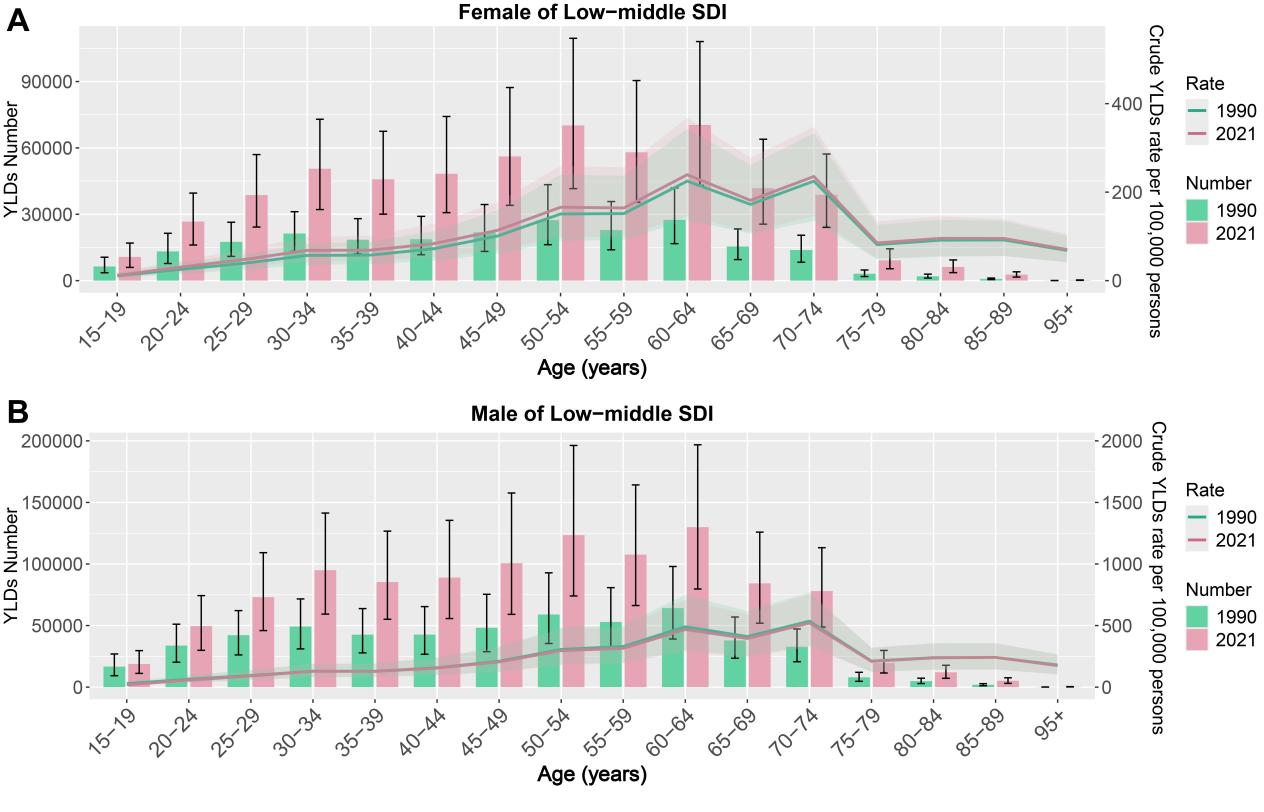
**

**Supplementary Figure S1(4). Burden of ONIHL among different genders and ages in Low-middle SDI region**

**
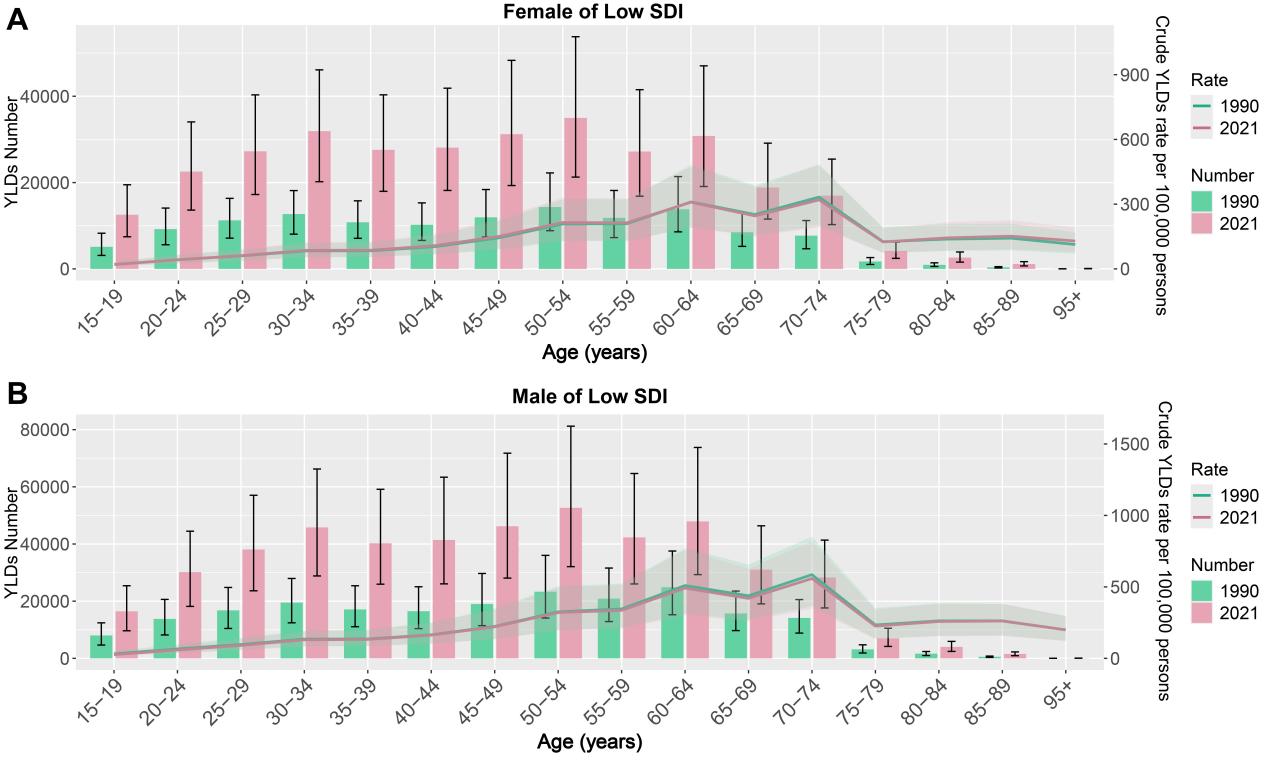
**

**Supplementary Figure S1(5). Burden of ONIHL among different genders and ages in Low SDI region**


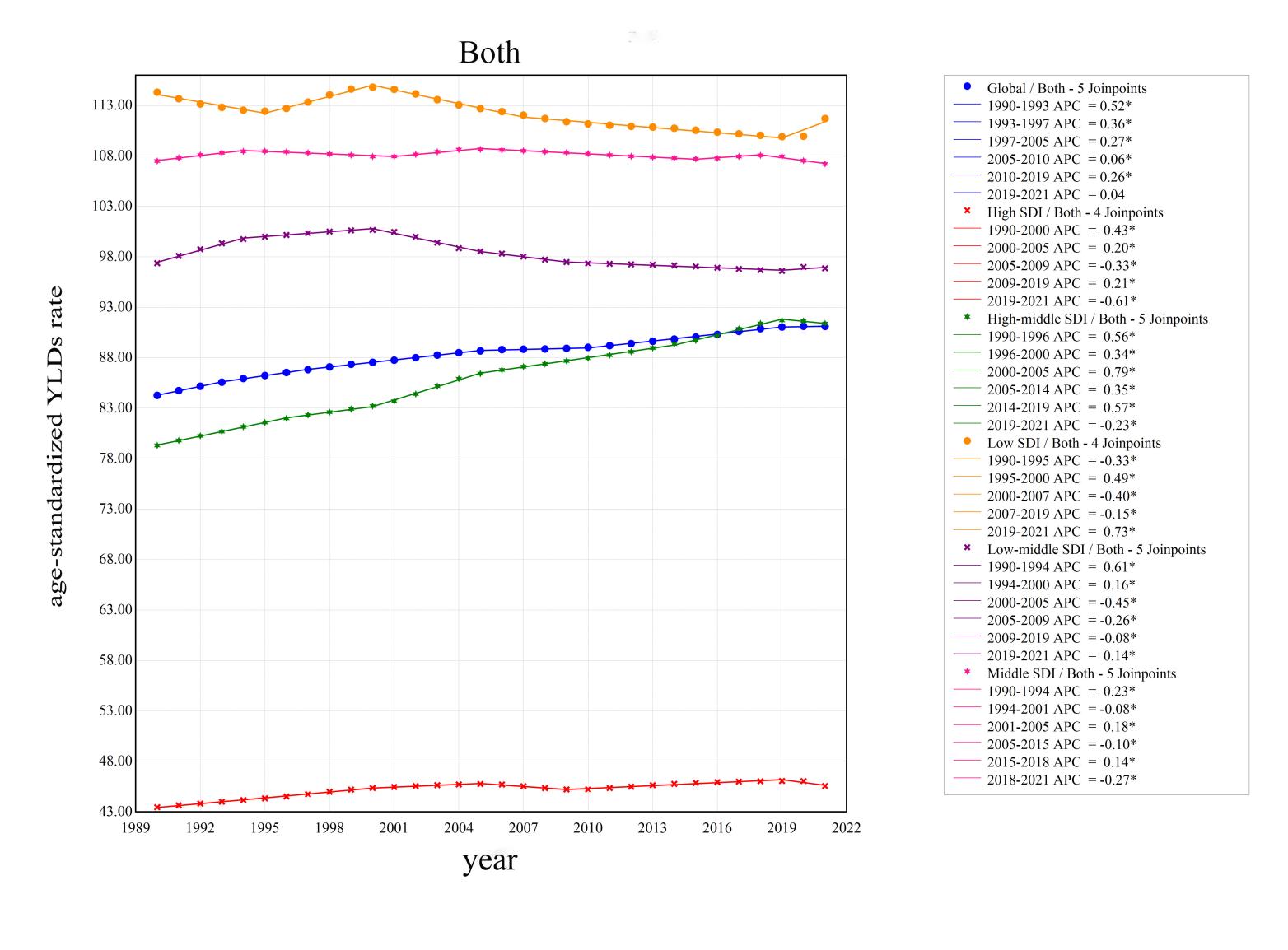
**Supplementary Figure S2.Trends of age-standardized YLDs rate in both female and male of ONIHL in SDI region**

APC, annual percent change

**Supplementary Table S2.Global, SDI Quintile Region female’s and Male’S age-standardized YLDs AAPCs from 1990 to 2021**

| Location | Global | | Female | | Male | |
| --- | --- | --- | --- | --- | --- | --- |
|  | AAPC | P-Value | AAPC | P-Value | AAPC | P-Value |
| Global | 0.25(0.24,0.26) | 0.00 | 0.44(0.43,0.45) | 0.00 | 0.13(0.12,0.14) | 0.00 |
| High SDI | 0.16(0.13,0.19) | 0.00 | 0.32(0.30,0.35) | 0.00 | -0.01(-0.05,0.03) | 0.71 |
| High-middle SDI | 0.46(0.45,0.47) | 0.00 | 0.61(0.60,0.62) | 0.00 | 0.32(0.30,0.34) | 0.00 |
| Middle SDI | -0.01(-0.09,0.00) | 0.07 | 0.06(0.05,0.07) | 0.00 | -0.04(-0.05,-0.02) | 0.00 |
| Low-middle SDI | -0.02(-0.03,0.00) | 0.03 | 0.34(0.33,0.36) | 0.00 | -0.12(-0.14,-0.10) | 0.00 |
| Low SDI | -0.08(-0.1,-0.05) | 0.00 | 0.05(-0.11,0.21) | 0.56 | -0.12(-0.13,-0.11) | 0.00 |

AAPCs, average annual percent changes

**Supplementary Table S3.Changes in YLDs according to population-level determinants and causes from 1990 to 2021**

| **Location** | **overall difference** | **change due to population-level determinants(% contribute to the total changes)** | | |
| --- | --- | --- | --- | --- |
|  |  | **Ageing** | **Population** | **Population Epidemiological change** |
| Both | | | | |
| **Global** | 3952909 | 843540.14(21.34%) | 2680479.5(67.81%) | 428889.331(10.85%) |
| **SDI** |  |  |  |  |
| **High SDI** | 277051.6 | 95893.38(34.61%) | 151228.5(54.58%) | 29929.708(10.8%) |
| **High-middle SDI** | 789003.7 | 253728.07(32.16%) | 352926.5(44.73%) | 182349.093(23.11%) |
| **Middle SDI** | 1568119.6 | 556739.1(35.5%) | 1025652.2(65.41%) | -14271.694(-0.91%) |
| **Low-middle SDI** | 873020.4 | 118750.7(13.6%) | 757023.6(86.71%) | -2753.861(-0.32%%) |
| **Low SDI** | 443849.3 | -15996.61(-3.6%) | 473624(106.71%) | -13778.094(-3.1%) |
| Female | | | | |
| **Global** | 1629954.9 | 320307.25(19.65%) | 1020493.83(62.61%) | 289153.839(17.74%) |
| **SDI** |  |  |  |  |
| **High SDI** | 104195.9 | 30951.93(29.71%) | 50635.33(48.6%) | 22608.648(21.7%) |
| **High-middle SDI** | 340845.9 | 100463.25(29.47%) | 139853.38(41.03%) | 100529.299(29.49%) |
| **Middle SDI** | 655506.4 | 230780.23(35.21%) | 411859.25(63.83%) | 12866.914(1.96%) |
| **Low-middle SDI** | 341909.6 | 44719.31(13.08%) | 252345.76(73.8%) | 44844.548(13.12%) |
| **Low SDI** | 186735.3 | -3189.37(-1.71%) | 186145.36(99.68%) | 3779.351(2.02%) |
| Male | | | | |
| **Global** | 2322954.1 | 532659.82(22.93%) | 1657333.1(71.35%) | 132961.184(5.72%) |
| **SDI** |  |  |  |  |
| **High SDI** | 172855.7 | 69806.35(40.38%) | 104088.4(60.22%) | -1039.042(-0.6%) |
| **High-middle SDI** | 448157.8 | 159593.59(35.61%) | 215634.3(48.12%) | 72929.928(16.27%) |
| **Middle SDI** | 912613.2 | 324040.92(35.51%) | 607414.3(66.56%) | -18842.014(-2.06%) |
| **Low-middle SDI** | 531110.8 | 67368.94(12.68%) | 497540.9(93.68%) | -33798.98(-6.36%) |
| **Low SDI** | 257114 | -14588.83(-5.67%) | 286429.5(111.4%) | -14726.689(-5.73%) |


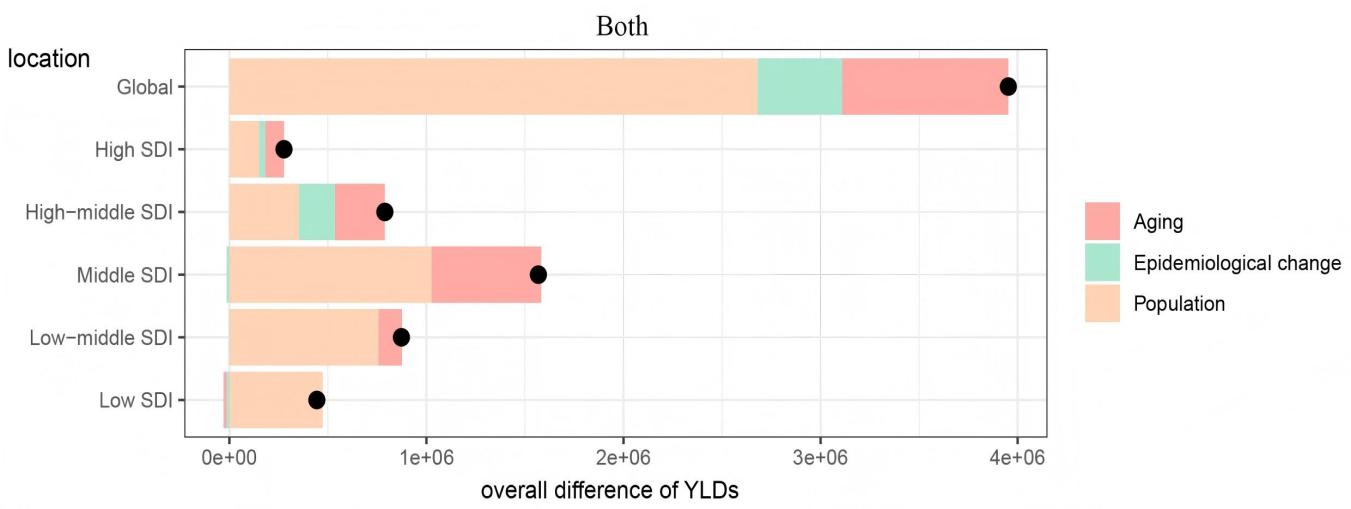


**Supplementary Figure S3.Changes in ONIHL YLDs(Both) according to population-level determinants of aging, population growth, and epidemiological change from 1990 to 2021 at the global level and by SDI quintile**


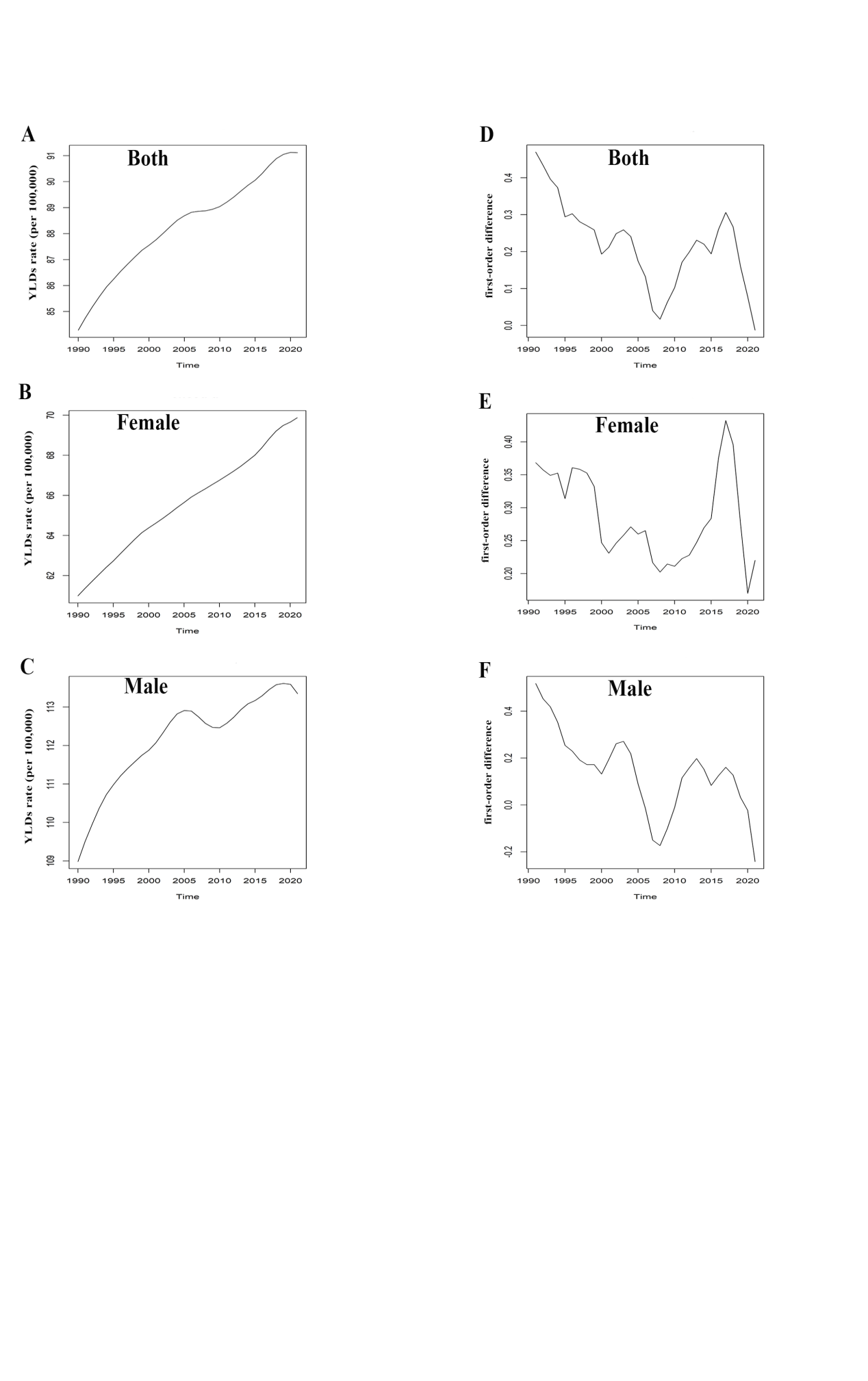


**Supplementary Figure S4.Timing diagram of ONIHL YLDs rate (A-C: Age-standardized YLDs rate of ONIHL; D-F: Age-standardized YLDs rate of ONIHL after first-order differencing)**


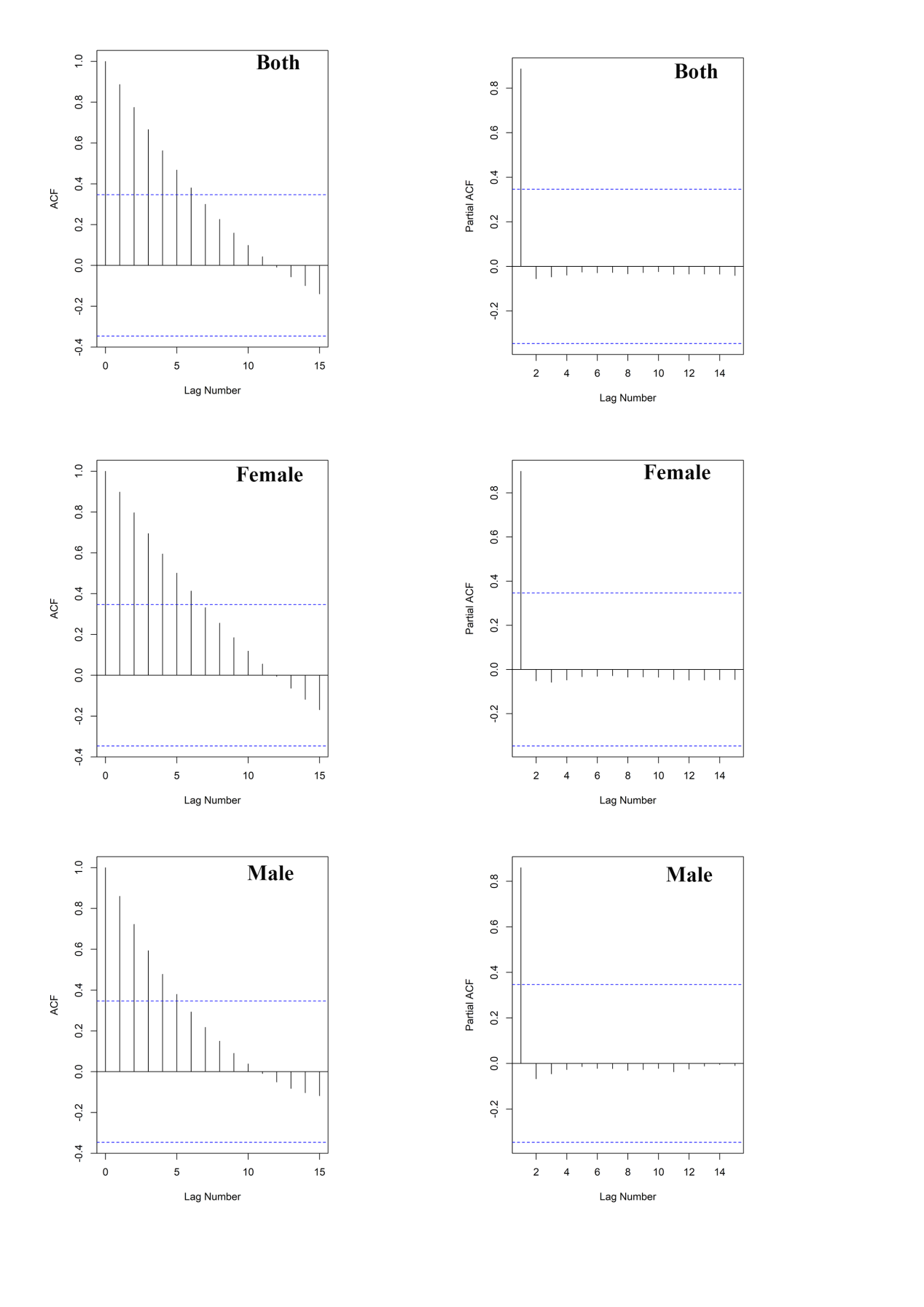


**Supplementary Figure S5. Autocorrelation function and partial autocorrelation function graphs of original data**


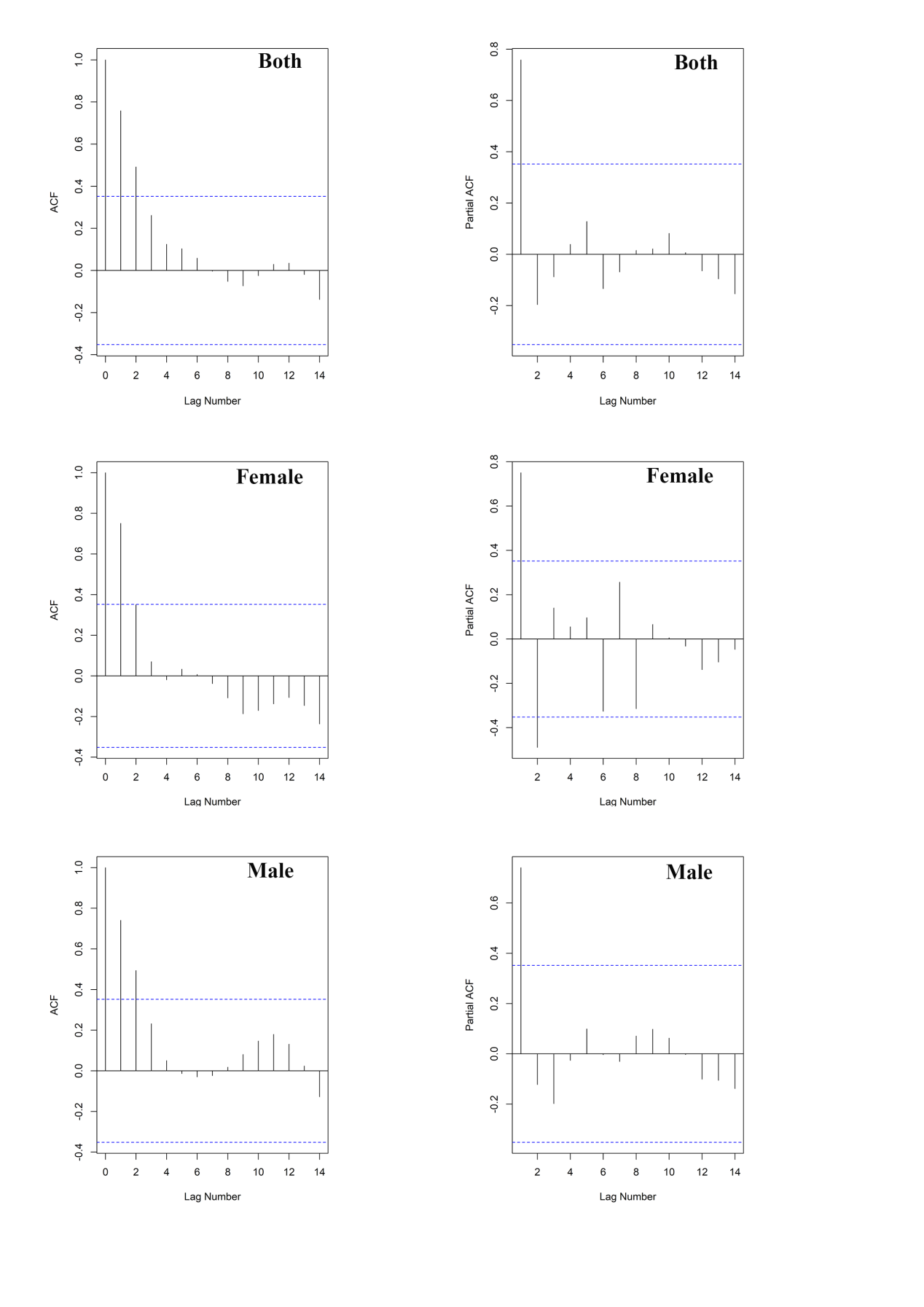


**Supplementary Figure S6. Autocorrelation function and partial autocorrelation function graphs after one order differencing**


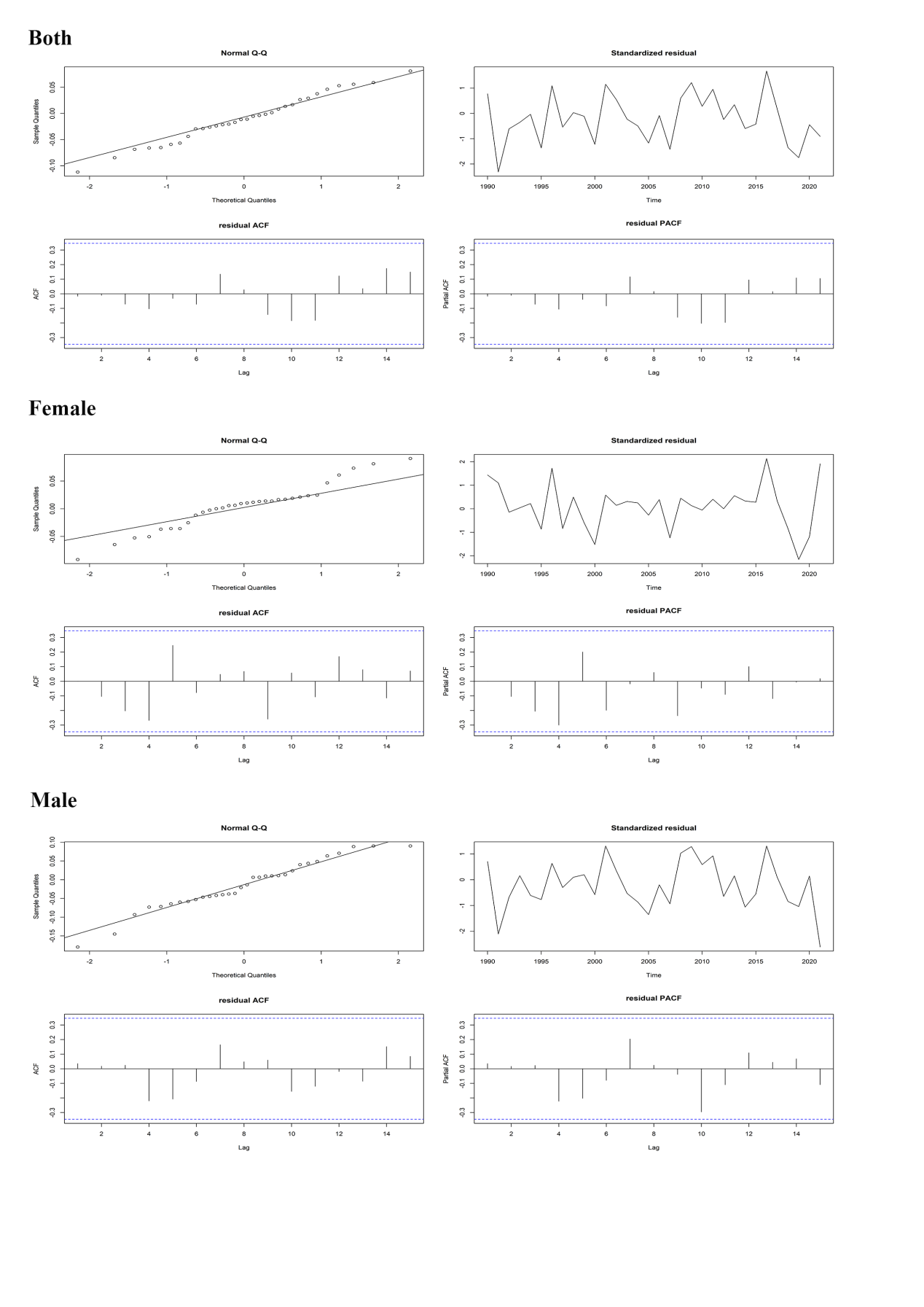


**Supplementary Figure S7.Residual Q-Q plots, Standardized residual,autocorrelation function and partial autocorrelation graphs of ARIMA models**

**Supplementary Table S4. The white noise test for ARIMA models**

|  | Ljung-Box λ^2^ | df | p-value |
| --- | --- | --- | --- |
| Both | 101.13 | 12 | 3.331e-16 |
| Female | 110.6 | 12 | 2.2e-16 |
| Male | 79.992 | 12 | 4.142e-12 |

**Supplementary Table S5. The predictive capacity of ARIMA models**

|  | ME | RMSE | MAE | MPE | MAPE | MASE |
| --- | --- | --- | --- | --- | --- | --- |
| Both | -0.010 | 0.046 | 0.037 | -0.011 | 0.042 | 0.167 |
| Female | 0.005 | 0.04 | 0.03 | 0.007 | 0.046 | 0.106 |
| Male | -0.014 | 0.066 | 0.053 | -0.013 | 0.047 | 0.284 |

ME: Mean Error, RMSE: Root Mean Squared Error, MAE: Mean Absolute Error, MPE: Mean Percentage Error, MAPE: Mean Absolute Percentage Error, MASE: Mean Absolute Scaled Error


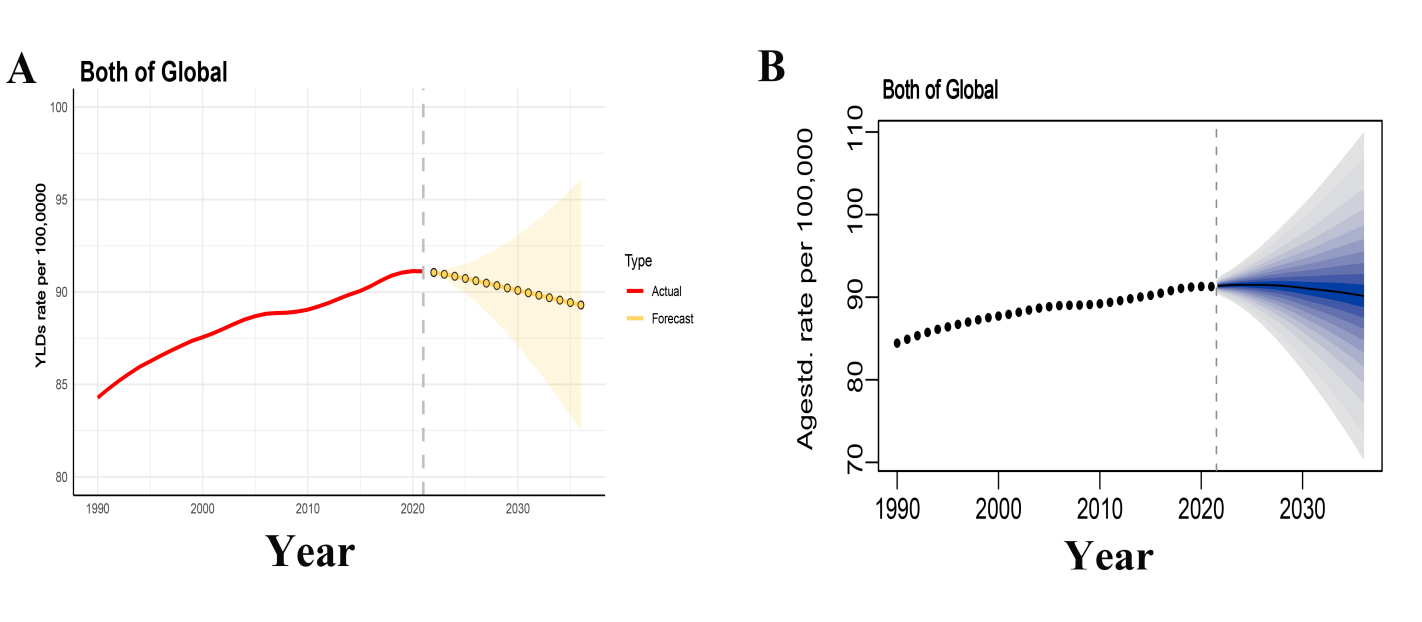


**Supplementary Figure S8.Prediction of ONIHL burden(Both)in Global from 2022 to 2036**

**(A:ARIMA model, the yellow lines represent the predicted trend and the light-yellow shaded regions represent the 95% confidence interval of predicted values; the gray dot vertical line split data into true value (1990-2021) and predicted value (2022-2036).B:BAPC model )**
